# Cryo-Electron Microscopy and Biochemical Analysis Offer Insights Into the Effects of Acidic pH, Such as Occur During Acidosis, on the Complement Binding Properties of C-Reactive Protein

**DOI:** 10.3389/fimmu.2021.757633

**Published:** 2021-12-16

**Authors:** Dylan P. Noone, Tijn T. van der Velden, Thomas H. Sharp

**Affiliations:** Department of Cell and Chemical Biology, Leiden University Medical Center, Leiden, Netherlands

**Keywords:** cryoEM, CRP, complement, structural biology, ELISA, acidosis

## Abstract

The pentraxin family of proteins includes C-reactive protein (CRP), a canonical marker for the acute phase inflammatory response. As compared to normal physiological conditions in human serum, under conditions associated with damage and inflammation, such as acidosis and the oxidative burst, CRP exhibits modulated biochemical properties that may have a structural basis. Here, we explore how pH and ligand binding affect the structure and biochemical properties of CRP. Cryo-electron microscopy was used to solve structures of CRP at pH 7.5 or pH 5 and in the presence or absence of the ligand phosphocholine (PCh), which yielded 7 new high-resolution structures of CRP, including pentameric and decameric complexes. Structures previously derived from crystallography were imperfect pentagons, as shown by the variable angles between each subunit, whereas pentameric CRP derived from cryoEM was found to have C5 symmetry, with subunits forming a regular pentagon with equal angles. This discrepancy indicates flexibility at the interfaces of monomers that may relate to activation of the complement system by the C1 complex. CRP also appears to readily decamerise in solution into dimers of pentamers, which obscures the postulated binding sites for C1. Subtle structural rearrangements were observed between the conditions tested, including a putative change in histidine protonation that may prime the disulphide bridges for reduction and enhanced ability to activate the immune system. Enzyme-linked immunosorbent assays showed that CRP had markedly increased association to the C1 complex and immunoglobulins under conditions associated with acidosis, whilst a reduction in the Ca^2+^ concentration lowered this pH-sensitivity for C1q, but not immunoglobulins, suggesting different modes of binding. These data suggest a model whereby a change in the ionic nature of CRP and immunological proteins can make it more adhesive to potential ligands without large structural rearrangements.

## Introduction

C-reactive protein (CRP) is a pentameric protein of 115 kDa composed of five identical non-covalently associated pentraxin domains (1, 2). To date, crystal structures of CRP display quasi-5-fold symmetry, with each monomer arranged in a toroidal manner, forming a disc with two distinct faces; the activating face (A-face) and binding face (B-face) (3). The B-face binds to a range of ligands, with the canonical example being phosphocholine (PCh) head groups in lipids such as phosphatidylcholine. This is a calcium-dependent interaction, with each CRP monomer chelating two adjacent Ca^2+^ ions. The A-face modulates many effector functions of CRP, such as activation of the classical complement pathway, where binding of complement component C1q to CRP is mediated *via* predominantly electrostatic interactions between the Ca^2+^-containing globular head domain of C1q (gC1q) and the A-face of CRP (4–8).

CRP is an acute phase protein, which is upregulated by the liver in response to proinflammatory stimuli such as interleukin 6 (IL-6), during which the serum concentration of CRP can increase by more than 1,000-fold to over 500 µg/ml (>4.3 µM) (9–11). Furthermore, CRP is found at sites of inflammation and injury (12, 13), possibly indicating a critical role for CRP in these regions. The microenvironment of inflamed tissues is characterized by elevated levels of reactive oxygen species and acidosis (14–21), with evidence that these factors modulate the biochemical properties of CRP, including ligand binding and complement activation (22–25). Furthermore, several structurally-distinct forms of CRP have been hypothesised to exist, suggesting that factors such as local acidosis could induce a structural rearrangement within CRP, thereby facilitating binding of complement components and affecting complement activation (26–28).

Here, we investigate the effect of different pH conditions associated with acidosis on CRP using high-resolution cryo-electron microscopy (cryoEM) and biochemical assays. We were able to determine 7 new structures of CRP in 4 different conditions; specifically, at pH 7.5 or pH 5, with and without PCh. Both pentameric and decameric structures were observed, with the pentamer to decamer ratio strongly affected by solution conditions, which enabled high-resolution structure determination of CRP decamers in all four of the conditions, and structure determination of pentamers in three of the conditions. These new models indicate that no large structural changes occur in the fluid phase. Density corresponding to protonation of histidine residues was observed, as well as a conserved water molecule adjacent to the disulphide bridge, with potential consequences for cysteine reduction. Furthermore, small movements of protonatable residues were observed, that may lead to an increase in the freedom of movement between subunits at low pH. Enzyme-linked immunosorbent assays (ELISA) showed a clear increase in ligand binding at pH 5, as well as increased binding to immobilised C1q and immunoglobulins. Overall, this study provides a model whereby subtle changes in charged residues on CRP and immunological ligands can markedly increase their association in response to inflammatory stimuli, potentially priming CRP for downstream immunological effector functions.

## Materials and Methods

### Chemicals and Proteins

All chemicals were purchased from Sigma-Aldrich (USA) unless otherwise specified. CRP and 50 kDa spin filters were purchased from Merck (236608 and UFC505024 respectively, USA). Mouse polyclonal anti-human CRP antibody was purchased from R&D Systems (MAB17074, USA). Rabbit anti-BSA antibody was purchased from Thermofisher (A11133, USA). Human IgG was kindly supplied by Leendert Trouw (IgG1, Alemtuzumab, LUMC). All other antibodies were purchased from Dako (Denmark) including, goat anti-mouse and anti-rabbit antibodies conjugated to horseradish peroxidase (P044701 and P044801 respectively) and rabbit IgG (anti-human C3, A0062). Complement component C1q was purchased from Complement Technologies (USA). A single-chain globular head domain of C1q (gC1q) (29), containing a C-terminal strep tag (protein sequence in **Supplementary Material**) was produced by U-Protein Express BV (Netherlands) in HEK293E-253 cells. ELISA plates were purchased from Thermofisher (C96 Maxisorp Nunc_Immunoplate, Thermofisher, USA). CryoEM grids, C-flat holey carbon 400 mesh grids with holes sizes of 1.2/1.3 µm or 2 µm, were purchased from Aurion (Netherlands). Negative stain EM grids, 200 mesh continuous carbon EM grids, were purchased from (Electron Microscopy Sciences, USA). Uranyl formate was obtained from SPI-Chem (16984-59-1, USA).

### Enzyme-Linked Immunosorbent Assays

ELISAs were performed using 96 well microtiter plates coated with 100 µl of ligand (gC1q, C1q, human IgG, rabbit IgG, HSA or BSA) at 10 µg/ml in 0.1 M sodium carbonate (pH 9.3). For coating controls, only the sodium carbonate solution was added. These were incubated for 1 hour at 37°C, then washed 3 times in phosphate buffered saline (PBS) with 0.05% Tween-20. Subsequently, wells were blocked with 100 µl of 0.1 M spermidine, before being incubated and washed as previously described. Next, 50 µl CRP was added at the concentration ranges and buffer conditions described in the figures. Briefly, CRP (1 mg/ml in 20 mM Tris, 140 mM NaCl, 2 mM CaCl_2_, 0.05% NaN_3_, pH 7.5) was diluted in either HEPES buffer at pH 7.5 (20 mM HEPES, 140 mM NaCl) or acetate buffer at pH 5 (140 mM NaAcetate, 40 mM Acetic Acid, 140 mM NaCl). Concentrations of calcium chloride were 2 mM, 50 µM or 0 mM, the latter also containing 5 mM EDTA, which were incubated for 1-2 hours at 37°C before washing. To detect CRP binding, 50 µl of a mouse polyclonal anti-human CRP antibody was added at 2 µg/µl, incubated for 1 hour, and washed as described above. Thereafter, 50 µl of a goat anti-mouse antibody conjugated to horseradish peroxidase was added and then incubated for 1 hour and washed. Absorbance at 415 nm was measured 15 minutes after addition of 50 µl of 2.5 mg/ml 2,2’-azino-bis(3-ethylbenzothiazoline-6-sulfonic acid) (ABTS) in citric acid buffer (0.15 M, pH 4.2) with 0.015% (v/v) H_2_O_2_ using a Clariostar plus plate reader (BMG Labtech, Germany). The different Ca^2+^ conditions were analysed separately. Each condition consisted of three independent triplicates for each ligand. All values were normalized to 1500 nM of CRP pH 5 binding to gC1q, with the values of the 0 nM CRP in each condition subtracted as background. For the BSA control plate, the same protocol as the 2 mM Ca^2+^ group was repeated with the ligand and BSA concentrations indicated in the figures. For detection, 50 µl of a rabbit anti-BSA antibody and later 50 µl of a goat anti-rabbit antibody were used. Positive controls were used to normalise the data from each independent replicate and then 0 nM BSA conditions were subtracted as background. Positive controls consisted of coating with BSA 20 µg/ml in 0.1 M sodium carbonate (pH 9.3) to 96 well plates and detected as described above for BSA binding to the classical complement pathway.

### CryoEM Sample Preparation and Data Collection

CRP (1 mg/ml in 20 mM Tris, 140 mM NaCl, 2 mM CaCl_2_, 0.05% NaN_3_, pH 7.5) was concentrated in a 50 kDa molecular weight cut-off spin filter to concentrations between 5-7 mg/ml. This step was also used to buffer exchange CRP to either pH 7.5 or pH 5 (20 mM Tris, 140 mM NaCl and 2 mM CaCl_2_ or 140 mM NaAcetate, 40 mM Acetic Acid,140 mM NaCl and 2 mM CaCl_2_ respectively). Tween-20 was added to a final concentration of 0.05% (w/v) and, if applicable, PCh to a final concentration of 2 mM. For sample preparation, multiple conditions were tried. Optimal results were obtained using 3 µl of buffer-exchanged CRP added to freshly glow discharged C-flat holey carbon 400 mesh grids with holes sizes of 1.2/1.3 µm or 2 µm, which were blotted for 3 s with Whatman No. 1 filter paper on a Leica EMGP, with a chamber at 4°C and 65% humidity, before being plunge frozen in liquid ethane.

Gain normalised mrc stacks (mrcs format) were collected on a Titan Krios microscope (ThermoFisher) at the Netherlands Centre for Electron Nanoscopy operated at 300 keV and equipped with a Gatan K3 detector and Gatan BioQuantum K3 energy filter with a slit width of 20 eV. Movies were acquired using a total dose of 50 e/Å^2^ with 50 frames, at 105,000× magnification with a calibrated pixel size of 0.836 Å and a defocus range of -0.8 to -2.0 µm.

### CryoEM Data Analysis

For all conditions, motion correction and dose weighting of movies was performed using MotionCor2 (30) as part of the implementation within Relion 3.1 (31). For the conditions without PCh, unweighted summed images were used in CTFFIND4.1 (32) to determine the CTF (box size 512 pixels and maximum resolution of 3 Å). Micrographs were manually inspected, and CTF fits with a confidence of less than 90% were discarded. Structures with PCh were motion corrected as described above and the CTF was estimated using Gctf (33) before manually inspecting the fits. Subsequently, poor CTF fits or micrographs that contained crystalline ice were discarded. Particles were picked from motion-corrected micrographs using the neural network within EMAN2 (34). All subsequent steps were carried out in Relion 3.1 and described in detail in the **Supplementary Material**. Briefly, for each condition, particle coordinates were imported, binned 4× and extracted with a box size of 64 pixels. The particles were subjected to 2D classification and classes representing contamination or noise were discarded. Next, 3D classification was used to select particles representing pentameric CRP, using a low pass filtered (40 Å) map of CRP [protein data bank (PDB) code 1B09] (2) as a reference map and used to generate a loose mask in Relion. These were then re-extracted with 2× binning and a box size of 128 pixels. These were again subjected to 2D classification to eliminate remaining contamination, poorly aligned particles or decamers. A final round of 3D classification was then used to generate 6 classes using the low pass filtered map of 1B09. Low resolution classes were discarded, before the remaining particles were refined with C5 symmetry, subjected to Bayesian polishing and refined again. These particles were extracted without binning and with a box size of 360 pixels. These were then refined with C5 symmetry imposed and thereafter subject to three iterations each of beam tilt, anisotropic magnification and per particle defocus. Refinement was performed with C5 symmetry before another round of polishing. Duplicate particles were removed, as defined by particles within 100 Å of each other, and a final refinement, using either C5 symmetry and C1 symmetry, the latter using a loose mask, was performed. To extract particles representing decamers, unmasked 3D classification was run on the 4× binned 2D classes using the low passed version of 1B09 as a reference map as stated above. Identified particles were extracted with a box size of 128 pixels with 2× binning. Next, 2D classification was used to remove poorly aligned particles, contamination or remaining pentamers. The resulting particles were refined with C1 symmetry using the best 3D class from the previous step as a reference map and then extracted without binning with a box size of 360 pixels. This was again refined with C1 symmetry, using the previous refinement as a reference map and subsequently subject to three iterations each of beam tilt, anisotropic magnification and per particle defocus. These particles were then refined with C1 symmetry and then polished. The polished particle set was then refined with C1 symmetry. Duplicates were then removed, as defined as particles within 140 Å of each other, before a final refinement without symmetry applied. Local resolution for both the pentameric and decameric maps was calculated using the implementation within Relion.

### Model Building

To build the model for pentameric CRP at pH 7.5, a monomeric subunit from a crystal structure of CRP (PDB code 1B09) (2) was placed into the cryoEM map as a rigid body using UCSF ChimeraX (35). Next, the model was manually adjusted using ISOLDE (36). Copies of this monomer model were then placed into the remaining 4 sites as rigid bodies before refinement using ISOLDE was repeated for the pentameric structure. The resulting pentameric model was automatically refined using Phenix real-space refine (37), setting the starting model from ISOLDE as a reference. The output from Phenix was manually inspected and refined in ISOLDE before a final round of real-space refinement in Phenix was performed with additional constraints on rotamer states. The resulting pentameric model was used to build decameric CRP at pH 7.5. Two copies of pentameric CRP were placed in the density map as rigid bodies before refinement using ISOLDE and Phenix real-space refine as before. These two structures (pentameric and decameric CRP at pH 7.5) were used as starting models of the remaining cryoEM maps. PCh was parametrised using eLBOW (38) and ISOLDE and placed in the maps as rigid bodies before refinement. For each map, manual refinement in ISOLDE and automatic refinement in Phenix was performed as described above. Figures were prepared using UCSF ChimeraX (35).

### Structural Analysis

Angles between each CRP monomer were compared from models derived from X-ray crystallography to those derived from our cryoEM data. Angles were measured between Asp60, an amino acid that coordinates one of the B-face Ca^2+^ ions, and the Asp60 residues on two neighbouring monomers using UCSF Chimera (39). This was repeated for each CRP monomer within both isolated pentamers and also each pentamer within the decamer or crystallographic unit cell. Root-mean-square deviation (RMSD) and cross correlation values between each model or map were calculated using UCSF Chimera (39). Bond lengths were measure in UCSF Chimera for pentameric and decameric cryoEM derived models, as well as PDB entries containing pentamers, 1B09, 3PVO and 1GNH (1–3), and two containing offset decamers, 3L2Y and 3PVN (3, 40). Monomer-monomer ionic bonds were measured between the amino acids indicated in the figures for each monomer. For Arg116–Glu42–Lys119, the average of both bonds present in this interaction were taken as the bond length at this position. Averages of each position at each interface per pentamer were also calculated. Pentamer-pentamer ionic bonds were measured in an identical manner, and averaged where multiple decamers were present in the unit cell.

## Results

### Ligand Binding to CRP A- and B-Faces Is Mediated by pH and Calcium

**Figure 1:**
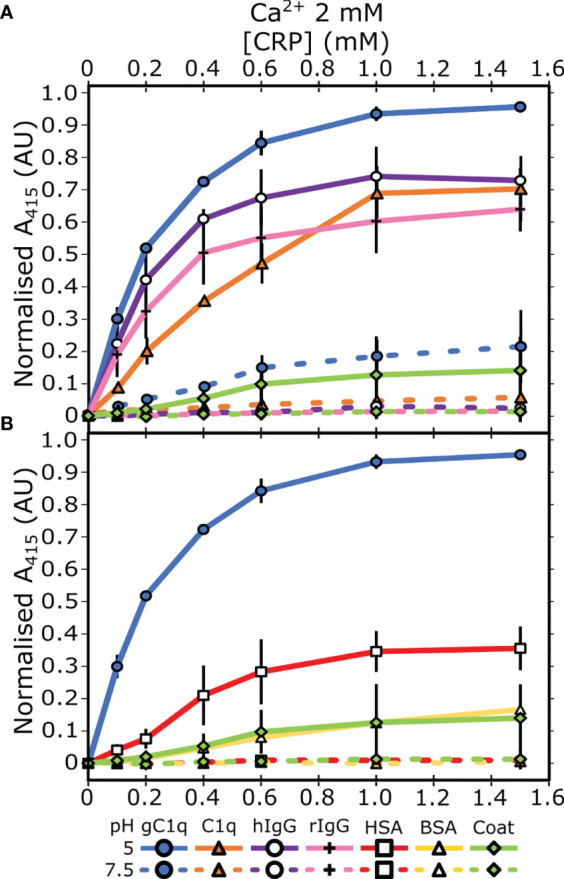
Measuring CRP binding to protein ligands at pH 7.5 and pH 5 in the presence of 2 mM calcium. **(A)** Binding of CRP to immobilised C1q, gC1q, and human and rabbit IgG (hIgG and rIgG, respectively). **(B)** Binding of CRP to immobilised HSA and BSA. CRP binding to gC1q at pH 5 and the spermidine coat are present in both panels for reference. Error bars show the standard deviation of 3 independent replicates.

Previous reports demonstrated that CRP has different biochemical properties at acidic pH (22–25), which may represent a mechanism whereby CRP exhibits increased binding to immunological proteins. To test this hypothesis, ELISAs were utilized to assess CRP association with proteins involved with activation of the classical complement cascade, specifically gC1q, C1q, and IgG. Both human serum albumin (HSA) and bovine serum albumin (BSA) were also tested to determine whether pH-induced binding was non-specific. Plates were coated with protein ligands before CRP was added at a concentration range of 0 – 1500 nM, with 2 mM Ca^2+^ at either pH 7.5 or pH 5. Minimal binding to all proteins was observed at pH 7.5. However, enhanced CRP binding at pH 5 was observed, where gC1q showed the greatest CRP binding (**Figure 1A**), with full-length C1q, and human and rabbit IgG (hIgG and rIgG, resp.) showing similar binding profiles (**Figure 1A**). Partial CRP binding was seen for HSA at pH 5, although this was below all immunological proteins, and all other serum proteins showed minimal binding (**Figure 1B**). To determine if increased binding at pH 5 was specific to CRP, we assessed binding of BSA to gC1q and C1q (**Figure S1**). Nominal binding was detected, even at higher (3 mM) concentrations, for either pH condition.

**Figure 2:**
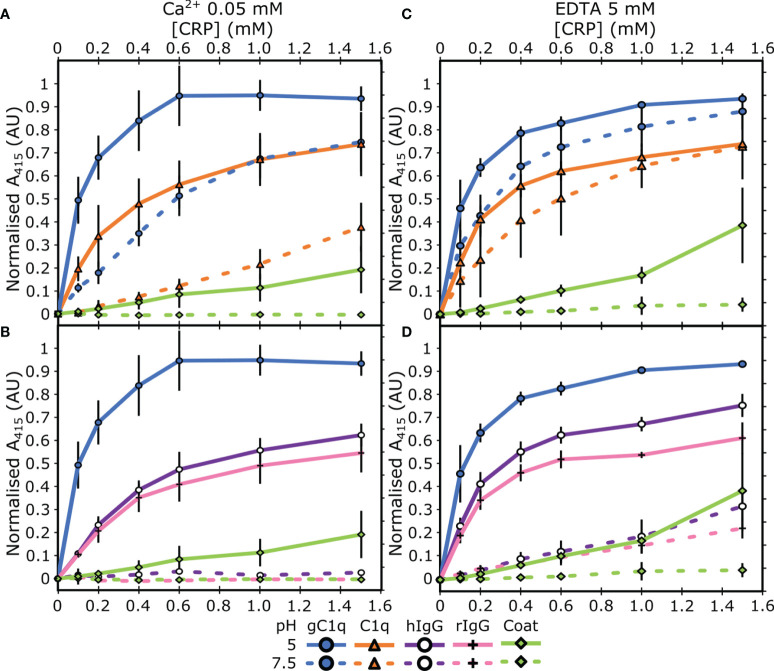
Assessing the influence of calcium on the binding properties of CRP. **(A, B)** Measuring CRP binding at 50 µM Ca^2+^ to **(A)** C1q and gC1q, **(B)** human and rabbit IgG. **(C, D)** Measuring CRP binding at 0 mM Ca^2+^ and 5 mM EDTA to **(C)** C1q and gC1q, **(D)** human and rabbit IgG. Binding is measured at pH 7.5 and pH 5. CRP binding to gC1q at pH 5 and spermidine (coat) is present in all panels for reference. Error bars show the standard deviation of 3 independent replicates.

Next, we assessed the link between this pH dependent binding relationship with the concentration of Ca^2+^. ELISAs were repeated with two different concentrations of Ca^2+^; 50 µM or 0 mM supplemented with 5 mM EDTA. C1q interacted with CRP, *via* the gC1q head groups, at pH 5 in a calcium-independent manner (**Figures 2A, C**), indicating that the B-face Ca^2+^ ions are not required for binding to these proteins (6–8). The differences between the binding at pH 7.5 and pH 5 of both gC1q and full-length C1q is reduced as the Ca^2+^ concentration is decreased (**Figures 1A** and **2A, C**). In contrast, for IgG, the variation in Ca^2+^ concentration had no effect on the pH dependence of the interaction with CRP, with all three Ca^2+^ concentrations displaying clear binding at pH 5, and minimal binding at pH 7.5 (**Figures 1A** and **2B, D**). In contrast to all of the above, serum albumin proteins exhibited very low levels of binding in each condition tested (**Figure S2**).

### CryoEM Reveals pH-Induced Structural Changes of CRP

**Figure 3:**
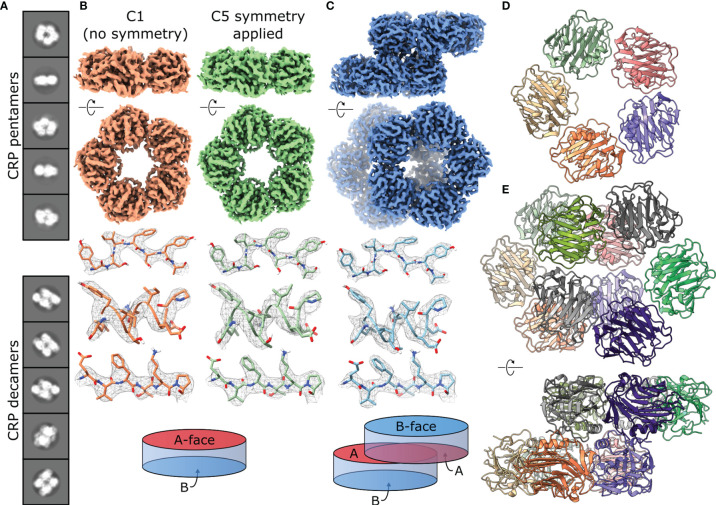
CryoEM of CRP reveals the co-existence of pentameric and decameric structures. **(A)** Class averages show pentameric and decameric structures in solution within the same sample (pH 7.5). **(B)** EM maps of CRP pentamers solved at pH 7.5 with both C1 (orange) and C5 (green) symmetry applied. Examples of sidechain density are shown above a schematic of the CRP pentamer as a disk with 2 opposing A and B faces. **(C)** EM map of a CRP decamer solved at pH 7.5, with examples of sidechain density and a schematic showing A-face stacking between the dimers of pentamers. **(D)** Top view of the atomic model of the CRP pentamer solved at pH 7.5. **(E)** Atomic model of the CRP decamer solved at pH 7.5, shown from the top and side. Panels **(B, C, E)** show both side and top-down views of pentameric and decameric maps or models related to one another *via* a 90° rotation.

To examine whether the pH-induced effects revealed by our biochemical analysis above had a structural basis, we used cryoEM to image four conditions of CRP; ± 2 mM PCh at pH 7.5 or 5. CRP exhibited adhesiveness to the grid, itself and to the air-water interface, which was improved by the addition of 0.05% Tween 20. Several grid types were tested, with C-flat holey carbon grids (1.2 µM hole size, 1.3 µM spacing and 400 mesh) giving optimal particle distribution. Whilst the addition of PCh negatively affected the particle distribution (**Figure S3**), we were able to collect more micrographs to partially compensate for the reduced number of isolated particles (**Table 1**). With these conditions, imaging on a 300 kV Titan Krios microscope yielded micrographs showing particles corresponding to CRP suspended in vitreous ice within the carbon holes (**Figure S3**). For each condition, 2D classification showed the presence of the expected CRP pentamers, as well as a variable portion of CRP decamers (**Figures 3A** and **S4**). The ratio of pentamers to decamers for each condition is shown in **Table 1**, as judged from the particle contributions from the respective final refinements, except for in the case of pentamers at pH 5 +PCh, where this was judged by final 2D classes. Both pH and the presence of PCh appeared to affect these ratios, as well as particle orientations within the vitreous ice (**Figures S3, S5**), with the pH 5 buffer producing more decamers compared to pH 7.5. The presence of PCh exaggerated the ratio at pH 5, almost entirely eliminating pentamers from the pH 5 +PCh condition; only 23,302 pentamers were detected as judged by 2D classes averaging, approximately 6.7% of the total number of CRP particles in this condition (**Table 1**). This prevented high resolution structure determination of a pentamer in this condition, although it was also likely hampered by suboptimal particle distribution caused by CRP aggregating in solution (**Figure S3**). In contrast, there was a slight increase in the prevalence of pentamers at pH 7.5 when in the presence of PCh.

**Table 1:** Parameters and validation statistics for cryoEM data collection, reconstruction refinement and corresponding atomic models.

Data collection							
Microscope	Titan Krios						
Camera	Gatan K3 detector						
Magnification	×105,000						
Voltage (kV)	300						
No. of frames	50						
Electron exposure (e−/Å^2^)	50						
Defocus range (µm)	-0.8 to -2.0						
Pixel size (Å)	0.836						
**Data processing**							
Condition	pH 7.5		pH 5		+PCh, pH 7.5		+PCh, pH 5
No. of micrographs	2,332		3,131		4,599		9,520
No. of total particles	650,386		740,919		932,224		1,805,017
Structure	Pentamer	Decamer	Pentamer	Decamer	Pentamer	Decamer	Decamer
EMPD	13467	13456	13471	13470	13469	13468	13472
PDB	7PKB	7PK9	7PKG	7PKF	7PKE	7PKD	7PKH
Symmetry imposed	C5 (C1)	C1	C5 (C1)	C1	C5 (C1)	C1	C1
No. of final particles	256,289	204,354	171,942	362,064	259,507	167,957	323,169
Decamer: Pentamer Ratio	0.56	0.44	0.32	0.68	0.61	0.39	0.93*
Map resolution (FSC = 0.143) (Å)	3.2 (3.5)	2.8	3.3 (4.0)	2.8	3.3 (3.5)	3.3	3.0
Map resolution range (Å)	3.1 – 3.6	2.8 – 3.8	3.2 – 3.8	2.6 – 3.5	3.3 – 3.6	3.2 – 4.0	2.8 – 3.6
Map sharpening B factor (Å^2^)	-107	-58	-115	-72	-125	-86	-72
**Refinement**							
Model resolution (FSC = 0.5) (Å)	3.1 (3.4)	2.8	3.3 (3.9)	2.8	3.2 (3.9)	3.2	3.0
Model composition							
No. of nonhydrogen atoms	8,175	16,350	8,175	16,350	8,230	16,460	16,490
No. of protein residues	1,030	2,060	1,030	2,060	1,030	2,060	2,060
No. of ligands	10	20	10	10	15	30	30
No. of waters	5	10	5	10	5	10	10
B-factors (Å2)							
Protein	74.33	47.52	86.13	46.4	60.3	46.67	50.78
Ligands	81.36	58.28	95.45	57.18	63.86	51.55	54.24
Water	66.56	40.78	77.85	37.04	53.43	39.95	42.21
RMSD							
Bond lengths (Å)	0.008	0.009	0.005	0.004	0.007	0.007	0.008
Bond angles (°)	0.905	1.064	0.856	0.902	0.854	0.857	0.962
Validation							
MolProbity score	1.53	1.81	1.6	1.55	1.32	1.35	1.46
Clash score	3.78	6.82	4.52	4.87	2.78	2.93	3.98
Rotamer outliers (%)	0	0	0	0	0	0	0
Ramachandran plot							
Favored (%)	94.8	93.38	94.51	95.69	96.27	96.08	95.93
Allowed (%)	5.1	6.27	5.49	4.26	3.73	3.92	4.07
Disallowed (%)	0	0.34	0	0.05	0	0	0

Values in parentheses for CRP pentamers are values for non-symmetrised (C1) maps and models. *The number of pentamers in this condition was calculated from the population of 2D classes, not the number of particles contributing to the final 3D maps.

The presence of pentamers and decamers necessitated 3D reconstruction of seven independent structures. Maps corresponding to pentameric CRP were solved with both C1 (no symmetry applied) and C5 symmetry (**Figures 3B** and **S6**). In all cases, applying C5 symmetry yielded maps of better resolution, corresponding to 3.2 – 3.3 Å (**Table 1** and **Figures S7, 8**). Although decamers appeared to adopt C2 symmetry (**Figure 3C**), they were solved without symmetry applied and reached resolutions of 3.3 – 2.8 Å (**Table 1** and **Figures S7, 8**). Each map showed clear side chain density (**Figures 3B, C** and **S9**), allowing us to build 10 models of CRP; pentamers with both C1 and C5 symmetry for all conditions except pH 5 +PCh, and decamers for all conditions (**Figure S9**). A crystal structure of CRP (PDB code 1B09) (2), was used as an initial structure to refine models of pentameric CRP at pH 7.5, in the absence of PCh, into both C1 and C5 symmetrised maps using Isolde and Phenix (36, 37), which was then used as a starting model for the other conditions. The resulting C5-symmetric pentameric CRP models for each condition were dimerised into the respective maps of decameric CRP as starting models for refinement.

**Figure 4:**
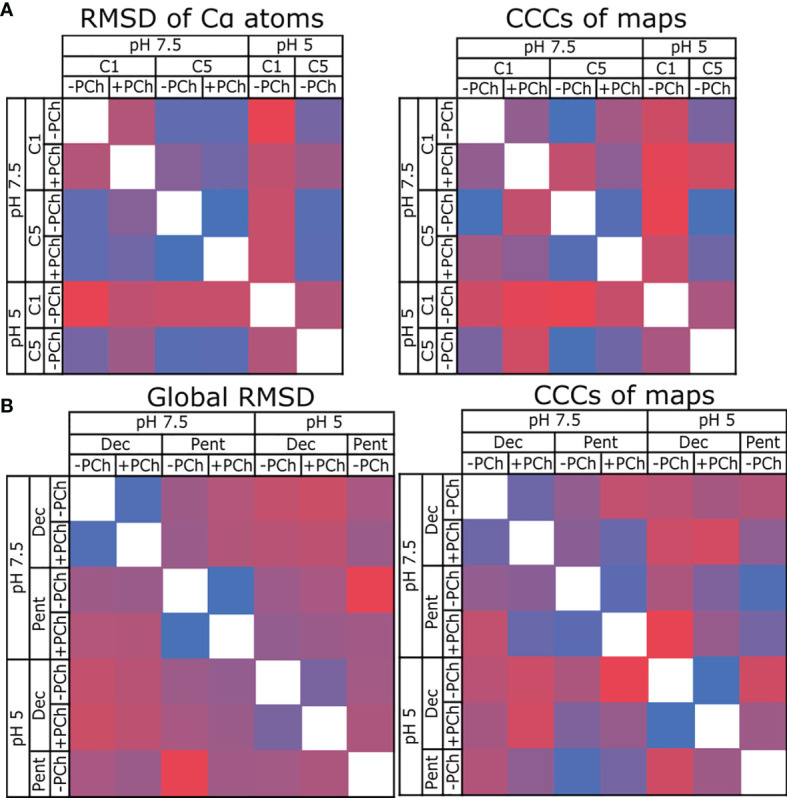
Comparing the similarity of CRP models and maps. **(A)** Comparing CRP pentamers refined into C1 and C5 symmetric maps at pH 7.5 and 5, ± PCh. RMSD is used to compare models, and cross-correlation coefficients (CCC) used to compare maps. **(B)** Comparing CRP decamers and pentamers at pH 7.5 and 5, ± PCh. In all panels, blue indicates high similarity (low RMSD, high CCC), red indicates divergence (high RMSD, low CCC).

Models of pentameric CRP built into either symmetrized or non-symmetrized maps showed no substantial differences (**Figure S10**), with RMSD values of the Cα backbone atoms of 0.525 Å (pH 5), 0.329 Å (pH 7.5) and 0.365 Å (pH 7.5 +PCh) (**Table 2** and **Figure 4A**), whilst cross-correlation calculations comparing the symmetrised and non-symmetrised maps also revealed a high degree of similarity, with coefficients of 0.953, 0.976 and 0.959, respectively. These low RMSD and high cross-correlation values both indicate highly similar structures. The increase in resolution upon map symmetrisation and high degree of similarity in the resulting maps and models strongly suggests that solution-phase CRP pentamers adopt C5 symmetry, which is reinforced by the low deviation in the angles between subunits in the C1-symmetrised pentameric models (**Table S1**). This was not apparent from many existing crystal structures, which displayed quasi-5-fold symmetry, as illustrated by the variable angles between subunits within each pentamer (**Table S2**) (1–3, 40, 41). Consequently, only CRP pentameric models derived from the higher-resolution C5-symetrised maps are discussed hereafter.

**Table 2:** Comparing C1 and C5 symmetric CRP pentamers in different conditions.

Condition compared	Cross correlation	Cα atoms RMSD
pH 7.5	0.9756	0.329
pH 7.5 +PCh	0.9593	0.365
pH 5	0.9529	0.525

Cross correlation and RMSD of protein backbone Cα were used to compare maps and models, respectively.

RMSD and cross-correlation coefficients comparing all decameric and pentameric models are shown in **Figure 4B** (see also **Table S3**). These data show that there is a high similarity between models and maps for each condition in both the presence and absence of PCh (e.g, pH 7.5 Decamer +PCh vs -PCh). Comparing pentamers -PCh at pH 7.5 vs pH 5, and pentamers +PCh at pH 7.5 vs pH 5, showed a small divergence in models (as measured by an RMSD of 0.72 Å) in both cases (**Table S3**), and the maps are highly similar, with high cross-correlation coefficients (0.9745 and 0.9659, respectively, **Table S3**). When cryoEM derived models were compared to CRP entries in the PDB – 1B09, 3PVO, 3LY2, 3PVN and 1GNH (1–3, 40) – a higher, but still small, global RMSD value was obtained (1.15 Å for cryoEM derived models compared to PDB models, and 0.72 Å for cryoEM models compared to each other). This indicates that there are no large differences between the models presented in this study and previous models resolved using X-ray crystallography (**Table S4**), which were all determined with a pH ≥7.

**Figure 5:**
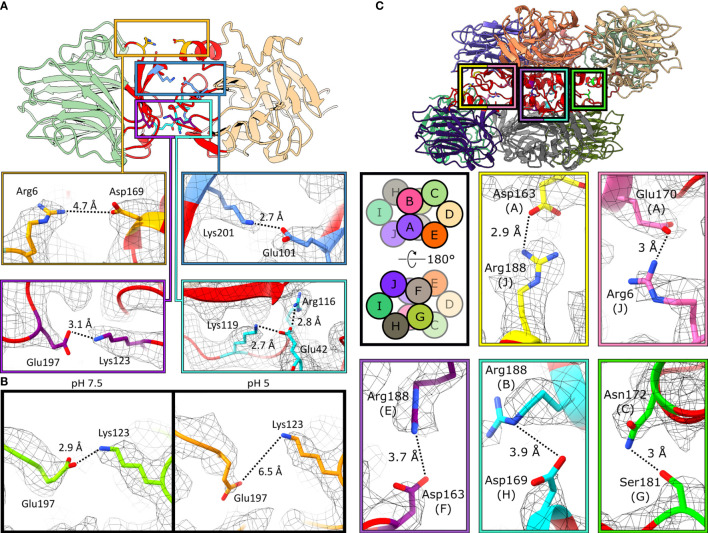
Monomers associate *via* ionic bonds within CRP pentamers and decamers. **(A)** Monomer-monomer interfaces are shown by the red regions. Five ionic bonds act as a zipper between subunits: Arg6–Asp169, Glu101–Lys201, Glu197–Lys123 and Arg116–Glu42–Lys119. Images are taken from C5 symmetric pentameric maps and models. **(B)** Glu197 shifts away from Lys123 at pH 5, breaking this ionic bond. Residues in green and orange represent pH 7.5 and pH 5, resp. Both are derived from the C5-symmetric pentameric models. **(C)** Pentamer-pentamer interfaces are shown in red. Five ionic bonds interact between pentamers. Schematic shows the subunit layout of the decameric models. Ionic bonds mediate pentamer-pentamer contacts. All images shown are from the pH 7.5 decamer.

CRP pentamers are composed of non-covalently associated subunits. Within the structures obtained by cryoEM, five intersubunit ionic bonds contribute to monomer-monomer interactions within the pentamer. These bonds form an ionic zipper from the A-face to the B-face of CRP (**Figure 5A**). These comprise, from the A-face to the B-face, of Arg6–Asp169, Glu101–Lys201, Arg116–Glu42–Lys119 and Lys123–Glu197 (**Figure 5A**). One of the salt bridges, Lys123–Glu197, is present at pH 7.5 but absent at pH 5 (**Figure 5B**).

Decameric CRP is formed from the offset stacking of two pentamers *via* their A-faces (**Figure 3C**). Intermolecular contacts between monomers within CRP decamers were also dominated by non-covalent ionic bonds, which formed a second ionic zipper (**Figure 5C**), perpendicular to that found in CRP pentamers (**Figure 5A**). Ionic bonds included Asp163(chain A)–Arg188(J), Glu170(A)–Arg6(J), Arg188(E)–Asp163(F), Arg188(B)–Asp169(H) and Asn172(C)–Ser181(G). Asp169 and Arg6 were able to form additional salt bridges in decamers compared to those formed in pentamers (**Figure 5A**), whilst still participating in monomer-monomer interactions. No major differences were observed between the decamers at different pHs, with or without PCh, other than the proportions of decamers to pentamers. Analysis of CRP, using a physiological concentration of 4.3 µM (0.5 mg/ml) (9) with the same buffering conditions used for cryoEM (pH 7.5 or pH 5, ± PCh), using size exclusion chromatography (SEC) resolved a single species, which internal protein calibrants allowed us to attribute to pentamers (**Figure S11A**). Applying dynamic light scattering (DLS) to measure the hydrodynamic diameter of more concentrated CRP solutions at pH 5 (~24 µM, ± PCh) also revealed a single species (**Figure S11B**). However, whilst negative stain electron microscopy showed that pentameric CRP was the dominant form of CRP at the buffers tested both for cryoEM and ELISA, there were small numbers of decamers observed in all conditions tested (**Figure S12**), even at a concentration of 6.7 µg/ml, lower than DLS (~2.8 mg/ml) and SEC (0.5 mg/ml).

**Figure 6:**
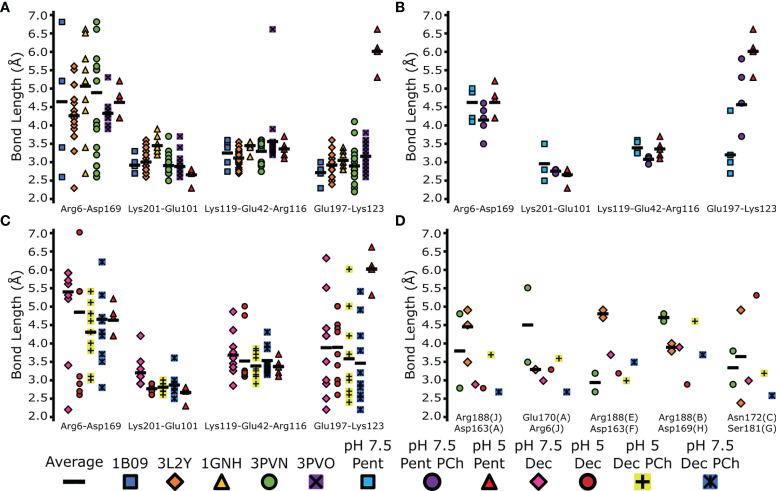
Comparison of inter-monomer and inter-pentamer ionic bond lengths in EM and X-ray derived models. The indicated bonds and interfaces within each model were measured and plotted (colored marks). Averages of these bonds were also calculated (black horizonal bars). **(A)** Comparison of the EM-derived pentameric CRP model at pH 5, and PDB depositions 1B09, 3LY2, 1GNH, 3PVO and 3PVN (1–3, 40). **(B)** Comparison of monomer-monomer ionic bonds between all pentameric CRP cryoEM-derived models. **(C)** Comparison of monomer-monomer ionic bonds between all decameric cryoEM derived protein models. Measurements from the pentameric CRP cryoEM-derived model at pH 5 is shown for reference in panels **(A–C)** for reference. **(D)** Comparison of interpentameric ionic bonds present in cryoEM-derived decameric models, and decameric CRP found in PDB entries 3LY2 and 3PVN. CryoEM derived decameric subunit labelling was used (A–J).

All intermonomeric bonds identified in our structures were also present in PDB entries 1B09, 3PVO, 3L2Y, 3PVN and 1GNH (1–3, 40), with similar bond lengths compared to pH 7.5 (**Figures 6A–C**). All PDB entries were obtained *via* X-ray crystallography with CRP at a pH ≥7.5 (**Table S5**). In agreement with our cryoEM data, Lys123–Glu197 in all crystal structures was shorter than pentameric CRP at pH 5 (**Figure 6A**), with an average bond length of 3.0 Å compared to 6.0 Å. Upon the addition of PCh to the CRP pentamer, Lys123–Glu197 adopted an intermediate bond length of 4.6 Å (**Figure 6B**). Intermonomeric ionic bonds found in decameric CRP at both pH 7.5 and pH 5 were similar to the crystal structures and the cryoEM-derived pentameric CRP at pH 7.5, except for at Glu197-Lys123 where there was a larger distribution of bond lengths, indicating conformational flexibility at this location (**Figure 6C**). It is also noteworthy that in all models there is a large variation in bond lengths between Arg6-Asp169, suggesting this bond is not formed between all subunits within each pentamer (**Figures 6A**–**C**). Decameric CRP is present in PDB entries 3LY2 and 3PVN (3, 40), and interpentameric ionic bonds identical to those in the cryoEM structures were identified (**Figure 6D**), which adopted a wider distribution of lengths that may indicate structural flexibility.

**Figure 7:**
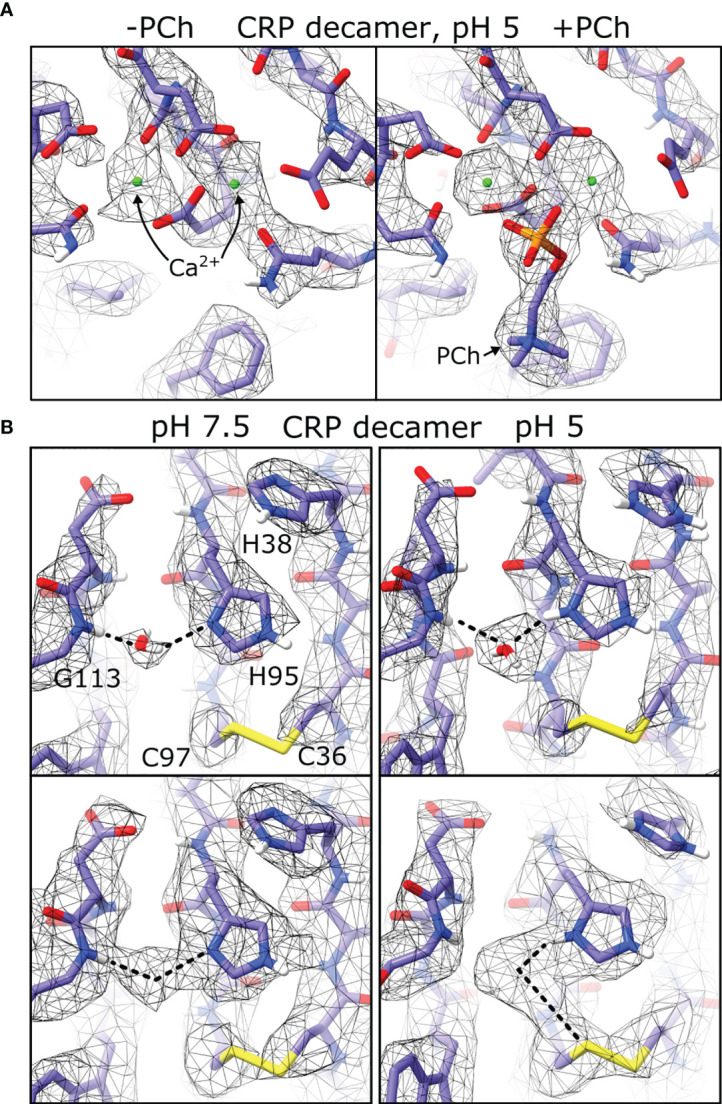
Differences in pH and ligand binding revealed by cryoEM. **(A)** Both Ca^2+^calcium binding sites shown in maps of CRP decamers at pH 5. Maps show density differences in the absence and presence of phosphocholine ligand (PCh). **(B)** Density corresponding to coordinated water molecules are present in both pH 7.5 and pH 5 (top), and display different density maps at higher isosurface thresholds (bottom).

In all maps of pentameric and decameric CRP, clear density was present for two Ca^2+^ ions (**Figure 7A**), and in conditions including PCh density for the ligand was visible and adopted similar conformations between the cryoEM derived models and those found in crystal structures (1–3, 40, 41). Strikingly, density was present adjacent to the imidazole ring of His95, the backbone amine of Gly113, and the disulphide bond formed from Cys36 and Cys97, which was not accommodated by the protein model (**Figures 7B** and **S13**). Density at this location was also present in two crystal structures of CRP (PDB codes 3L2Y and 3PVN) (3, 40) where it was modelled as a water molecule. This density therefore resolves the rotamer state of this residue, with the N1 imidazole nitrogen forming a hydrogen bond to the water molecule. This density shifted between His95-Gly113 at pH 7.5, and His95-Cys97 at pH 5, possibly due to protonation of His95 at low pH rearranging the hydrogen bonding network from the water hydrogen at pH 7.5 to the water oxygen at pH 5 (**Figure 7B**).

## Discussion

CRP binding and acidosis both occur locally at sites of inflammation (12, 13), and previous biochemical data provides evidence of pH-dependent modulation of CRP activity (22–25), which may relate to targeted complement activation and regulation. We explored the effect of pH on CRP binding, which showed a strong pH-dependent binding response to gC1q, C1q and IgG (**Figure 1A**). This was shown to be a specific effect of CRP, as BSA did not exhibit the same pH dependent binding to C1q or gC1q (**Figure S1**). Furthermore, CRP was shown to bind to these immunological ligands to a greater extent than non-immunological albumin proteins (**Figure 1B**), suggesting the change in pH modulates the immunological functions of CRP rather than simply representing non-specific binding. These observations may indicate a regulatory response, whereby CRP only binds to complement activating proteins and activates complement at sites of inflammation, where localised acidosis can reduce the pH of the surrounding milieu to as low as pH 5 (14–21). Previous studies have also shown that the ligand binding properties of CRP change in response to acidic pH (22). Here, we add to this hypothesis with the notion that acidosis specifically increases the association between CRP and complement activating proteins. How these pH-dependent binding relationships relate to complement activation and progression remain to be determined; however, it may be that at pH 5 CRP amplifies the immunological signal of complement activation, in a similar way to alternative pathway amplification by C3 (42), by binding to opsonins to allow for more efficient complement activation and signalling. Furthermore, given the potential array of functions CRP has in other aspects of the immune system, such as phagocytosis (7), testing more ligands for pH-dependent binding could reveal whether enhanced CRP binding to effector proteins at low pH is a global immunological phenomenon or only occurs with complement-related proteins and antibodies.

Many interactions with the B-face of CRP are calcium dependent (2), and so to provide insights into whether the B-face coordinated Ca^2+^ ions were involved in binding, we reduced the Ca^2+^ concentration in the two different pH buffers to 50 μM and 0 mM (supplemented with EDTA to chelate Ca^2+^ ions pre-bound to CRP). C1q binds to the A-face of CRP (6–8), and in keeping with that binding to C1q and gC1q at pH 5 was equivalent at 2 mM, 50 μM and 0 μM Ca^2+^ (**Figures 1**, **2**), indicating that this interaction is calcium-independent and therefore likely to be *via* the A-face. Similarly, the interaction between CRP and IgG was not modulated by Ca^2+^, suggesting the B-face Ca^2+^ is not needed in this interaction (**Figures 1**, **2**). However, the interaction between CRP and IgG remained highly dependent on pH regardless of Ca^2+^ concentration, with pH 5 always producing greater binding, whereas the pH difference between C1q and gC1q was decreased with a reduction in the concentration of calcium. This indicates that CRP may have a different binding mode to IgG compared to C1q, which is supported in the literature, as the pH-dependent association of CRP with ligands such as complement factor H did not result in the exposure of a cryptic CRP epitope, whereas binding to IgG did (22). Given the spatial and temporal proximity of both antibodies and CRP during infection and inflammation (13, 43), this provides more evidence for the notion that CRP not only initiates complement, but acts as an amplifier after localised activation at sites of acidosis.

Evidence has suggested that acidic environments and binding to lipid ligands cause structural changes in CRP, illustrated by the exposure of cryptic epitopes under certain conditions (23, 44). To date, all structures of CRP have been solved using crystallography, and each of these structures have been crystallized at pH ≥7.5 (1–3, 40, 41). To determine whether structural changes occur in acidic environments, we used single-particle analysis cryoEM to solve the first solution-phase structures of CRP, at pH 5 and pH 7.5 and in the presence and absence of PCh (**Figures 3** and **S6, S9**). CRP protein particles formed two distinct populations on the cryoEM grids, the expected pentamers as well as decamers formed from the offset stacking of two pentamers (**Figure 3**). The proportions of these varied between conditions (**Table 1**), with pH 5 containing more decamers than at pH 7.5, 68% vs 44% decamers, respectively. The addition of PCh amplified this difference, with pH 5 containing 93.3% decamers vs 40% at pH 7.5. This dimer-of-pentamers arrangement has been observed before and was described as either a crystallization artefact (1, 40) or a potential native structure (3). No crystallization is required for cryoEM using single-particle analysis, hence these decamers represent solution-phase structures of CRP, previously posited based on analytical ultracentrifugation (AUC) (45). These were not apparent in SEC at an acute phase concentration of CRP and were not detected even at higher concentration by DLS (**Figure S11**). This may indicate that the high concentrations of CRP used increased, or even caused, decamerization. However, decamers were also detected at all three concentrations of Ca^2+^ using negative stain EM, even at CRP concentrations much lower than those used in the biochemical assays (**Figure S12**). This may indicate a transient pentamer-decamer equilibrium in solution that is not detectable by SEC or DLS, but is by AUC (45). Our data shows that this equilibrium favours decamer formation at pH 5 and when bound to lipidic ligands such as PCh; consequently, at local sites of inflammation and acidosis, CRP bound to lipid membranes may also favour decamerization. Residues implicated in C1 binding and/or complement activation are predominantly located on the A-face (6–8) (**Figure S14**), and therefore binding of C1q to CRP will be sterically hindered by decamerization, which could inhibit complement activation by CRP at low pH. Conversely, it could be envisaged that CRP bound to two separate lipid membranes *via* B-face interactions could subsequently form a decamer *via* their A-faces, leading to agglutination of bacterial membranes or apoptotic blebs. Given pentraxins similarity to antibodies in terms of complement activation, it would be of interest to investigate whether they also share agglutination as an immune response with antibodies. Indeed, CRP is known to agglutinate lipid suspensions in a PCh and Ca^2+^ dependent manner (46), which has been linked to medically relevant conditions such as fat embolisms (47). These two effects, complement activation and agglutination, are not mutually exclusive and, analogously to IgG antibodies, both effects would enhance immune defence. Artificial decamerization of CRP, *via* the B-face, using a bivalent PCh analogue showed promise as a therapeutic to inhibit CRP binding and treat cardiovascular and inflammatory diseases (48). With these new data, it would be worthwhile to explore the therapeutic potential of CRP decamerization *via* the A-face, as demonstrated here.

We were able to reconstruct maps for all 4 decamers and pentamers in 3 of the conditions (**Figure S6**); the low proportion of pentamers at pH 5 in the presence of PCh hindered structure determination for that condition. Nevertheless, single-particle analysis yielded 7 high-resolution models of CRP (**Figures 3** and **S9**). In contrast to the large pH-mediated differences observed by ELISA, structural analysis revealed no large structural changes between pH 7.5 and pH 5 (**Figure 4B**). This indicates that the response of CRP to acidosis at inflammatory loci has a biochemical rather than a structural basis. Structural analysis of pentamers strongly suggests that pentameric CRP adopts C5 symmetry in solution (**Figure 4A**). Many previous structures of CRP adopted quasi-5-fold symmetry (1, 2, 40, 41) (**Table S2**). This variation in monomer contact angles highlights a flexibility at this non-covalent interface. Models of CRP pentamers derived from high-resolution C5-symmetric cryoEM reconstructions revealed that a series of ionic bonds mediate intersubunit interactions within pentamers (**Figure 5A**). However, one of the four intermonomer ionic bonds appears to break at pH 5, caused by a shift of Glu197 (**Figure 5B**), possibly affecting flexibility between subunits. This was reinforced by a similar trend being observed in previous X-ray structures, all of which were obtained at a pH ≥7.5 (**Figure 6** and **Table S5**). The addition of PCh resulted in an intermediate ionic bond length at this position. Hence, two factors that are known to prime CRP for complement activation, pH and PCh binding (9, 25), appear to effect this bond. Further work will be needed to determine whether this flexibility impacts downstream immunological functions, such as displaying binding sites for FcγR or C1q and the association with lipid membranes. The latter which may in fact result in further structural changes undetected in this study.

CRP binding to C1q and gC1q at pH 7.5 was modulated by the concentration of Ca^2+^ (**Figures 1**, **2**), which did not occur at pH 5. We hypothesised that changing the pH and concentration of Ca^2+^ was revealing the effect of the electrostatic environment around CRP and the protein ligands. The A-face has a large proportion of acidic and charged residues (49), so binding to this face may be susceptible to changes in the electrostatic environment. The pentamer-pentamer interface is regulated by salt bridges (**Figures 5C** and **6D**), and much more decamerization was observed at pH 5, supporting an interaction dominated by electrostatic bonds that was confirmed by the cryoEM imaging. Therefore, the propensity of CRP to decamerise under certain conditions may be taken as an indirect measure of the adhesiveness of the A-face. This is reinforced by the biochemical data that clearly shows that CRP binds to a greater extent to A-face ligands, gC1q and C1q, at pH 5 and at lower concentrations of Ca^2+^ (**Figures 1**, **2**). Taken together, these data indicate that an acidic environment, as well as lower concentrations of Ca^2+^, can increase association of A-face ligands, such as C1q.

CryoEM data also revealed a water molecule chelated by His95 and Gly113, adjacent to Cys97 of the disulphide bridge, in all conditions tested (**Figures 7B** and **S13**). A change in the electron density between His95, Gly113 and Cys97 indicates protonation of His95 at pH 5, but not at pH 7.5 (**Figures 7B**), and in both cases one of the water hydrogen atoms occupies space proximal to the disulphide sulphur atom, which may signify that this cysteine residue is primed for reduction. This may explain the observed synergistic effects of both ROS and pH on CRP, yielding a reduced CRP variant that is better able to activate the immune system (23), and further supports the suggestion that there is a subtle change in the biochemistry at this site, which could be important in the altered immunological properties of CRP at sites of inflammation. A water molecule is also found in other structures of CRP (3, 40), as well as serum amyloid P (SAP) (40, 50) and neuronal pentraxin (NPTX)-1 (51) where they are coordinated by the same residues. His95 and Gly113 (CRP numbering) are also conserved in PTX3, NPTX2 and NPTX-receptor (**Table 3**), and it is reasonable to predict that these (currently unknown) structures will also coordinate water, and may respond to ROS or pH changes in a similar way to CRP.

**Table 3:** Sequence alignment of human pentraxins.

CRP	EVTVAPVHICTSWESASGIVEFWVDGKPR	116
SAP	EKFPAPVHICVSWESSSGIAEFWINGTPL	114
PTX3	VSLGRWTHLCGTWNSEEGLTSLWVNGELA	273
NPTX1	INDGKWHHICVTWTTRDGVWEAYQDGTQG	313
NPTX2	VSDGKWHHICVTWTTRDGMWEAFQDGEKL	317
NPTXR	LKDNGWHHICIAWTTRDGLWSAYQDGELQ	379
	*:* * : .* : .*	

Positions of His95, Cys97 and Gly113 (CRP numbering) are shown in bold. Conserved sequences, and conservative and semi-conservative mutations are indicated by ‘*’, ‘:’ and ‘.’, respectively.

This work represents the first time that cryoEM has been applied to pentraxin biology. With data collected within 1 day per condition we were able to reconstruct maps with a resolution sufficient to see Ca^2+^ ions, water molecules and PCh ligand binding (**Figure 7**). This demonstrates the potential of cryoEM to enable much higher-throughput structural analysis of pentraxins with other, potentially novel, ligands, and other aspects of pentraxin biology that have a structural basis.

## Data Availability Statement

The cryoEM maps and associated models generated for this study were deposited in the EM database (EMDB) and protein database (PDB) using the following codes: decameric CRP at pH 7.5, EMDB-13456 and PBD-7PK9; pentameric CRP at pH 7.5, EMDB-13467 and PBD-7PKB; decameric CRP+PCh at pH 7.5, EMDB-13468 and PBD-7PKD; pentameric CRP+PCh at pH 7.5, EMDB-13469 and PBD-7PKE; decameric CRP at pH 5, EMDB-13470 and PBD-7PKF; pentameric CRP at pH 5, EMDB-13471 and PBD-7PKG; decameric CRP+PCh at pH 5, EMDB-13472 and PBD-7PKH.

## Author Contributions

DN performed all ELISAs, with help from TV. DN performed experiments, data analysis, cryoEM imaging and reconstructions. DN and TS analysed cryoEM data. TS built atomic models and supervised the work. DN and TS wrote the manuscript. All authors contributed to the article and approved the submitted version.

## Funding

This research was supported by the following grants to TS: European Research Council Grant 759517; The Netherlands Organization for Scientific Research Grants OCENW.KLEIN.291 and VI.Vidi.193.014.

## Conflict of Interest

The authors declare that the research was conducted in the absence of any commercial or financial relationships that could be construed as a potential conflict of interest.

## Publisher’s Note

All claims expressed in this article are solely those of the authors and do not necessarily represent those of their affiliated organizations, or those of the publisher, the editors and the reviewers. Any product that may be evaluated in this article, or claim that may be made by its manufacturer, is not guaranteed or endorsed by the publisher.
