# Supplementary material for: Cryo-Electron Microscopy and Biochemical Analysis Offer Insights Into the Effects of Acidic pH, Such as Occur During Acidosis, on the Complement Binding Properties of C-Reactive Protein
Source: Front Immunol. 2021 Dec 16;12:757633. doi: 10.3389/fimmu.2021.757633 (PMC8716467; doi:10.3389/fimmu.2021.757633)
Supplement: Supplementary file 1 [file DataSheet_1.pdf]

Supplementary Material

1 Supplementary Materials and Methods

Protein sequence for a single-chain gC1q protein.

A single-chain gC1q protein with the following sequence was synthesised by U-Protein Express BV (Netherlands) in HEK293E-253 cells. The signal peptide is in bold and cleaved off during secretion. The triple strep-tag is underlined.

MARPLCTLLLLMATLAGALAGSDQPRPAFSAIRRNPPMGGNVVIFDTVITNQEEPYQNHSGRFVCTVPGY
YYFTFQVLSQWEICLSIVSSSRGQVRRSLGFCDTTNKGLFQVVSGGMVLQLQQGDQVWVEKDPKKGHIYQ
GSEADSVFSGFLIFPSAGSGKQKFQSVFTVTRQTHQPPAPNSLIRFNAVLTNPQGDYDTSTGKFTCKVPG
LYYFVYHASHTANLCVLLYRSGVKVVTFCGHTSKTNQVNSGGVLLRLQVGEEVWLAVNDYYDMVGIQGSD
SVFSGFLLFPDGSAKATQKIAFSATRTINVPLRRDQTIRFDHVITNMNNNYEPRSGKFTCKVPGLYYFTY
HASSRGNLCVNLMRGRERAQKVVTFCDYAYNTFQVTTGGMVLKLEQGENVFLQATDKNSLLGMEGANSIF
SGFLLFPDMEAAAWSHPQFEKGAAWSHPQFEKGAAWSHPQFEKGAA

Size exclusion chromatography

CRP (1 mg/ml, Tris 20 mM, NaCl 140 mM, $CaCl_2$ 2 mM, sodium azide 0.05% (w/v), pH 7.5) was concentrated and buffer exchanged to 5.9 – 7.5 mg/ml into either Tris 20 mM, NaCl 140 mM, $CaCl_2$ 2 mM, pH 7.5 or NaAcetate 140 mM, Acetic Acid 40 mM, NaCl 140 mM, $CaCl_2$ 2 mM, pH 5, using 50 kDa cut off spin filters. This solution was then used to prepare 50 µl of 0.5 mg/ml of 4 different buffered conditions. These conditions used the pH 7.5 and 5 buffers described above, with or without 2 mM phosphocholine (PCh). CRP was diluted into the appropriate conditions and incubated for 1 hour at 37°C directly before injecting into a Superdex200 increase (s200I) 3.2/300 size exclusion column (SEC) (Cytiva, USA). Background was subtracted from the data and each condition was normalized to its maximum value.

Dynamic light scattering

CRP (1 mg/ml, Tris 20 mM, NaCl 140 mM, $CaCl_2$ 2 mM, sodium azide 0.05% (w/v), pH 7.5) was concentrated and buffer exchanged to ~5 mg/ml into NaAcetate 140 mM, Acetic Acid 40 mM, NaCl 140 mM, $CaCl_2$ 2 mM, pH 5. This was used to prepare a final solution containing ~2.8 mg/ml CRP with 0.05% Tween-20 with or without 2 mM PCh. The diameter of the particles in solution were measured using a DynaPro NanoStar (Wyatt Technology, USA) using a microcuvette (P/N: 162697-02, 1 µL – DPN, Wyatt technology, USA), and a technical triplicate was collected with the attenuator set to 50%.

Negative stain EM

CRP (1 mg/ml, Tris 20 mM, NaCl 140 mM, $CaCl_2$ 2 mM, sodium azide 0.05% (w/v), pH 7.5) was diluted to 6.7 µg/ml into either Tris 20 mM, NaCl 140 mM, pH 7.5 or NaAcetate 140 mM, Acetic Acid 40 mM, NaCl 140 mM, pH 5. The two different pH buffers also had either 2 mM $CaCl_2$, 50 µM $CaCl_2$ or 5 mM EDTA, resulting in 6 different conditions. The solutions were then incubated at 37°C for 2 hours, before 6 µl were applied to continuous-carbon EM grids (200 mesh EM grids, Electron Microscopy Sciences, USA) and incubated for 1 minute. Excess liquid was then removed with filter

paper (Whatman, Merck, USA), before 10 µl of uranyl formate (2%) was added to stain for 10 seconds, before excess stain was removed with filter paper. Samples were left to air-dry before being imaged on an FEI Tecnai 12 Twin Transmission electron microscope (ThermoFisher, USA) with a LaB_6 filament operating at 120 kV. Images were acquired with a defocus of ~0.5-1 µm on an Eagle 4k x 4K CCD camera (ThermoFisher).

CryoEM data analysis of CRP at pH 7.5 -PCh:

All reconstructions were performed using Relion (1). Particles were extracted to produce a stack of 650,386 particles that were binned 4× with a box size of 64 pixels. The particles were subject to 2D classification with 200 classes and a 110 Å soft circular mask. Classes representing bad particles (e.g. contamination or noise) were discarded yielding a stack of 495,078 particles. To select pentameric CRP, 3D classification was utilized, using a low pass filtered (40 Å) map of PDB deposition 1B09 as a reference map and mask. Classes that resembled the tetrameric like side view of the decamer were discarded yielding a stack of 367,537 particles. These were then re-extracted to 2× binning with a box size of 128 pixels. These were subject to 2D classification with 100 classes and a 110 Å soft circular mask. Bad particles or decamers were discarded producing a stack of 322,949 particles. 3D classification was then used with 6 classes, using the low pass filtered map of 1B09 as previously described. Low resolution classes were discarded producing classes representing 256,289 particles. They were then refined with C5 symmetry, subject to Bayesian polishing and refined again. These particles were extracted without binning and a box size of 360 pixels. These were then refined with C5 symmetry imposed and thereafter subject to beam tilt, anisotropic magnification and per particle defocus estimation within Relion 3.1 (1). This was repeated twice for a total of three iterations. A further C5 refinement was carried out, and then the particles were polished as before. Duplicates were removed, as defined by particles within 100 Å of each other, yielding 254,184 particles. This particle set was used for final refinements with both C5 symmetry and C1 symmetry that reached 3.2 Å and 3.5 Å respectively with the latter using a loose mask. To extract decamers from the population of particles, unmasked 3D classification was carried out on the 4× binned 2D classes. Classes representing decamers were selected yielding a stack of 204,452 particles. These were extracted with a box size of 128 pixels and binned 2×. The particles underwent 2D classification with 100 classes and a 110 Å soft circular mask. Bad particles, contamination or pentamers were discarded producing a set of 204,354 particles. This was refined with C1 symmetry and then extracted without binning with a box size of 360 pixels. This was again refined with C1 symmetry and subsequently subject to three iterations of beam tilt, anisotropic magnification and per particle defocus respectively as described above. These particles were then refined with C1 symmetry and then polished. The polished particle set was then refined with C1 symmetry. Duplicates were then removed, as defined as particles within 140 Å of each other, yielding a particle stack of 185,953. This was refined with C1 symmetry resulting in a final map of 2.8 Å. Local resolution for both the pentameric and decameric maps was calculated using an implementation within Relion.

CryoEM data analysis of CRP at pH 5 -PCh:

Particles were extracted with 4× binning and a box size of 64 pixels, from two data collection sessions, to give a combined total of 733,877 particles. These underwent 2D classification with 50 classes and a 110 Å soft circular mask. Bad classes and contamination were discarded, giving a stack of 617,674 particles. To filter out pentamers from the decamers in the population, masked 3D classification was utilized. Decamers were identified via a characteristic tetrameric like side view and discarded. This resulted in a particle set of 238,619 particles, which were thereafter extracted with 2× binning and a

box size of 128 pixels. This stack underwent 2D classification with 50 classes and a 110 Å soft circular mask. Bad classes and decamers were discarded producing a set of 207,193 particles. This was refined with C5 symmetry imposed and reextracted without binning and a box size of 360 pixels. This stack of particles underwent 2D classification with 50 classes and a 110 Å soft circular mask. Good classes were selected yielding a total of 198,659 particles. These later went into 3D classification with 4 classes. The lowest resolution class was discarded resulting in a set of 171,942 particles. This was refined with C5 symmetry and thereafter underwent three iterations of beam tilt, anisotropic magnification and per particle defocus estimation as described above. The particles were then polished and refined using C5 symmetry. Duplicates were then removed as defined by particles that were closer than 100 Å, resulting in a final stack of 171,144 particles. This was then refined with C5 symmetry 3.3 Å and C1 refinement with a soft mask 4 Å. To select the decamers unmasked 3D classification with 6 classes was used on the particle set form the 4× binned 2D classification. Classes representing decamers were selected resulting in a stack of 362,064 particles. These were extracted with 2× binning and a box size of 128 Å and subjected to 2D classification with 50 classes and a circular soft mask of 130 Å. All the classes were of good enough quality so were all used in a subsequent refinement with C1 symmetry. These were extracted without binning and a box size of 360 pixels before 2D classification was repeated with 50 classes and a soft circular mask of 140 Å. Again, all classes were of good quality so were used in a refinement with C1 symmetry. This particle set was then subjected to three iterations of beam tilt, anisotropic magnification and per particle defocus, respectively, as described previously. Thereafter the particles were refined with C1 symmetry imposed. The particles were polished and duplicates were removed, as defined as particles closer than 140 Å, to give a final stack of 315,988 particles. This was refined with C1 symmetry to give a final map of 2.8 Å. Local resolution for both the pentameric and decameric maps was calculated using an implementation within Relion.

CryoEM data analysis of CRP at pH 7.5 +PCh:

Particles were extracted with 4× binning and a box size of 64 pixels to give a total of 1,596,930 particles. The particles were subject to 2D classification with 200 classes and a 110 Å soft circular mask. Bad classes and contamination were discarded yielding a particle stack of 1,461,767 particles. These were subject to unmasked 3D classification using a low pass filtered map of 1B09 as a reference with 6 classes. A 3D class corresponding to a pentamer was selected to filter out this subpopulation of 414,369 particles. These were then extracted to 2× binning with a box size of 128 Å and subject to another iteration of 2D classification with 100 classes and a 110 Å soft circular mask. Bad classes, contamination and decamers were discarded, resulting in 397,433 particles. These particles were then used in an unmasked 3D classification run, using a low pass filtered map of 1B09 as before with 2 classes. The highest resolution class was selected, corresponding to 269,217 particles, and then refined with C5 symmetry imposed. The particles were then extracted without binning with a box size of 360 pixels. They were then refined with C5 symmetry imposed and then subject to polishing within Relion. The particles were refined again and then duplicates were removed, as defined by particles within 140 Å of each other, yielding 259,507 particles. Next, the C5-symmetric refinement was repeated, as well as a separate refinement using C1 symmetry coupled with a loose mask, yielding maps with resolutions of 3.3 Å and 3.5 Å respectively. To select decamers, a class representing a well-defined decamer was selected from the 4× binned unmasked 3D class averaging job previously described. This corresponded to 393,732 particles. These were reextracted with 2× binning and a box size 128 pixels. The particles underwent 2D classification with 100 classes and a 130 Å soft circular. Contamination, poorly aligned particles and pentamers were discarded, giving a total of 352,317 particles. This stack was used in a 3D classification job with 2 classes, using the decameric class from the previous 3D classification job as a reference map. The highest resolution class, containing 175,177 particles, was then extracted

without binning and a box size of 360 pixels. This was refined without symmetry and polished. The polished particles set was refined before duplicates were removed, as defined by particles within 140 Å of each other, yielding 167,957 particles. This was refined as before yielding a map of 3.3 Å. Local resolution for both the pentameric and decameric maps was calculated using an implementation within Relion.

CryoEM data analysis of CRP at pH 5 +PCh:

Given the suboptimal particle distribution for this condition, three separate data collections were required to acquire sufficient numbers of particles for high-resolution analysis. In the case of the decameric population of particles, all three data collections were processed independently until post particle polishing. For the first and second data collection, henceforth known as optics group 1 and 2, the same in-silico purification strategy was used, whilst keeping the data sets separate and independent. First, particles were extracted to 4× binning with a box size of 64 pixels. Particles underwent 2D classification with 100 classes and a 110 Å soft circular mask. Classes representing contamination or poorly aligned particles were discarded. These were used as an input for an unmasked 3D classification job with 4 classes, using a low pass filtered map of the PDB entry 1B09 as a reference map. A single class representing a decamer was selected. These were extracted with 2× binning with a box size of 128 pixels and were subject to further 2D classification with 100 classes and a 130 Å soft circular mask. Contamination, poorly aligned particles or classes representing pentamers were discarded. These were extracted with no binning and a box size of 360 pixels. These underwent another round of 2D classification, in an identical manner as described above, and well aligned decamers were selected giving a total of 99,026 and 119,214 particles for optics group 1 and 2, respectively. These were separately refined with no symmetry imposed and then subject to 3 iterations of a sequence of beam tilt, anisotropic magnification and per particle defocus estimation. For the third data collection, optics group 3, particles were extracted with 4× binning and subject to 2D class averaging as described above for optics groups 1 and 2. Particles were then subject to two rounds unmasked 3D classification using the same settings as for optics group 1 and 2. Contamination and low-resolution particles were discarded after each iteration, resulting in a stack of 1,086,837 particles. They were then re-extracted with 2× binning and a box size of 128 pixels and subject to 2D class averaging classification with 100 classes and a 130 Å soft circular mask. Both pentameric and decameric particles were selected, but contamination or poorly aligned particles were discarded yielding 983,286 particles. As before, 3D classification was used to discard particles comprising lower resolution classes and then the resultant particles underwent 2 iterations of 2D class averaging with a 130 Å soft circular mask. This resulted in a population of decamers and pentamers consisting of 858,265 particles. Decamers were selected yielding a stack of 471,385 particles which underwent 3D classification with 2 classes using a low pass filtered version of our pH 7.5 cryoEM derived decameric model as a reference map. The highest resolution class, comprising 378,258 particles, was selected and extracted without binning and a box size of 360 pixels. This was then refined without symmetry and subject to particles polishing. Subsequently, the particles were refined as before and beam tilt, anisotropic magnification and per particle defocus were estimated as described in previous conditions. This was then refined without symmetry. Subsequently, all three optics groups were combined, subject to 3D class averaging with 2 classes and the highest resolution class was selected and duplicates were removed, as defined by particles within 140 Å of each other, producing a final combined particle stack of 323,169 particles. This was refined without symmetry to yield a 3 Å map. To separate out the pentamers, 2D classes representing pentamers were selected from the 2D classification job at 2× binning for optics group 1. For optics group 2, a 3D class representing a pentamer was taken from the 4× binned data, subsequently

extracted with 2× binning and underwent 2D classification selecting for pentamers. Pentamers from optics group 3 were selected from the second iteration of 2D classification in the 2× binned data. This resulted in three independent stacks of 10,007, 49,289 and 385,487 particles for optics groups 1, 2 and 3, respectively. Particles were processed together hereafter. Particles were subjected to 2 iterations of 2D classification with 100 classes and a soft mask of 150 Å. Contamination or poorly aligned particles were discarded after each iteration, yielding a stack of 174,402 particles. The particles were underwent unmasked 3D classification, with 2 classes using a low pass filtered map of 1B09 as a reference. After, two iterations of 2D classification were used as before to further clean the particle set. Refinements were attempted several times during this cleaning process, with none reaching below ~8 Å in resolution. Instead, the best pentameric 2D classes from the final iteration of 2D classification were selected, then duplicates were removed, as defined by particles within 100 Å of each other, yielding 23,302 particles.

2 Supplementary Figures

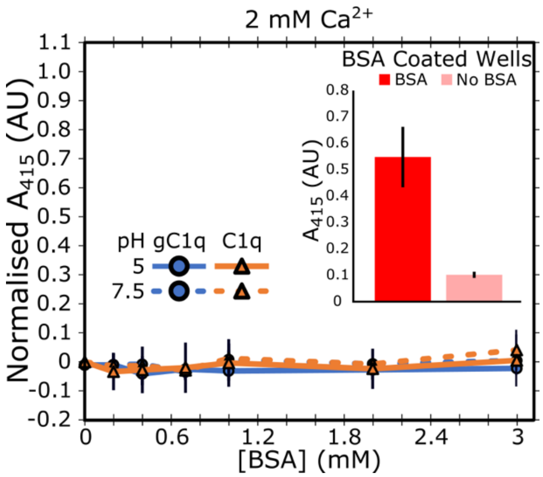

Figure S1. BSA does not show binding to C1q or gC1q at pH 7.5 or 5. Wells were coated with gC1q or C1q and later BSA was added at pH 7.5 to 5 in a concentration range from 0 – 3 mM. The inset bar chart shows signal from positive and negative controls, consisting of wells coated with BSA or no ligand respectively. Results of gC1q and C1q binding were normalized to positive controls. Error bars show the standard deviation of three independent replicates.

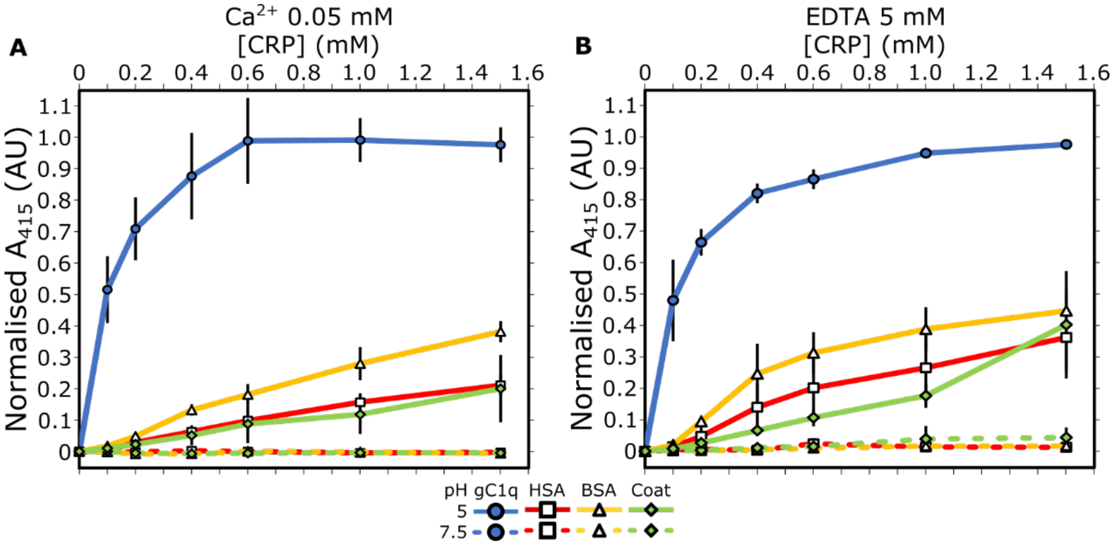

Figure S2. CRP displayed pH dependent but minimal binding to serum proteins at 50 µM (A) or 0 mM calcium (B). Wells were coated with BSA or HSA. Next, CRP was added at pH 7.5 or 5 at the concentrations indicated above. At 50 µM (**A**) only BSA at pH 5 showed binding above background. At 0 mM calcium and 5 mM EDTA (**B**) both BSA and HSA shows minimal binding above background.

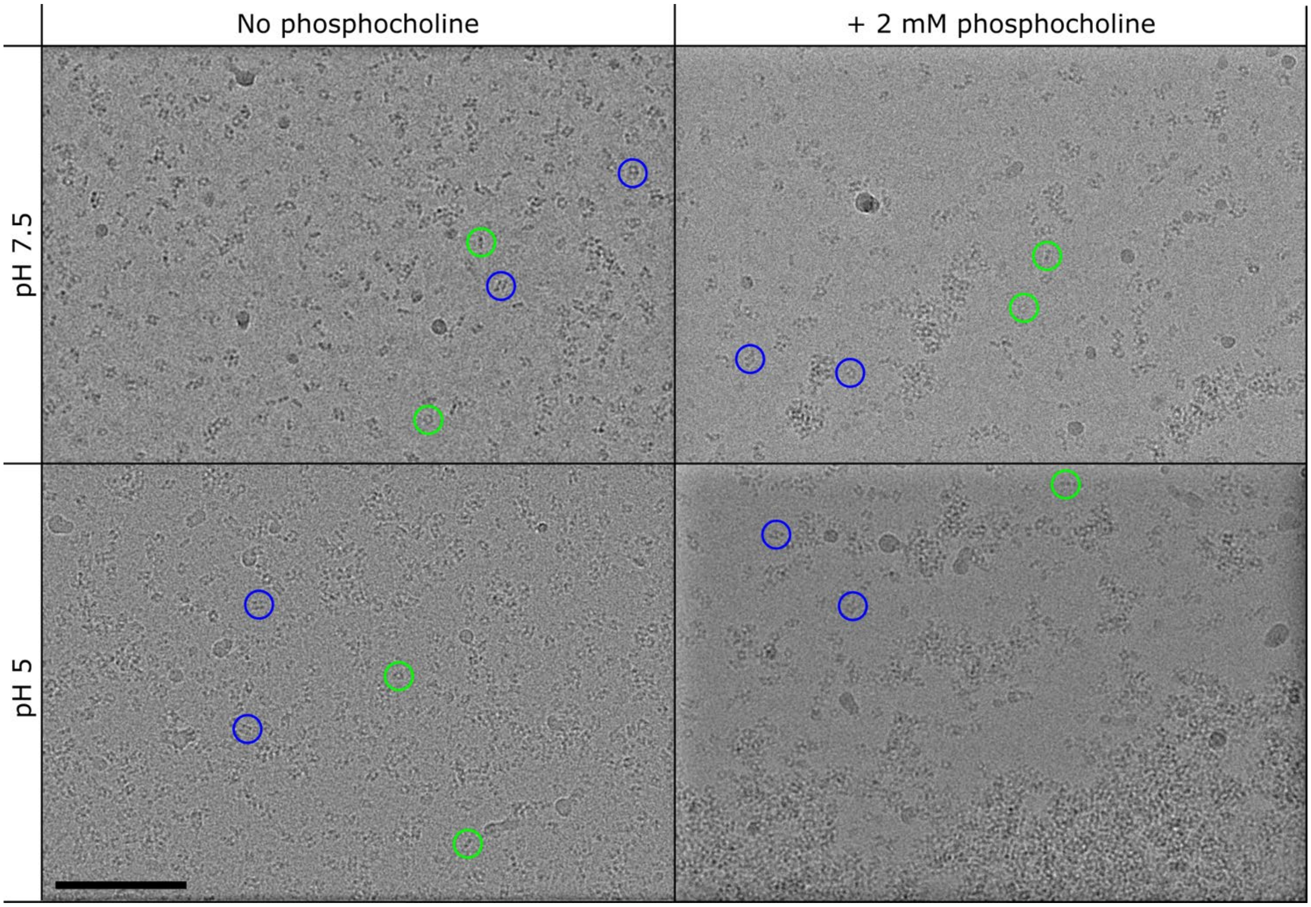

Figure S3. Representative micrographs of CRP suspended in vitreous ice for all 4 conditions. Decameric and pentameric CRP particles are identified with blue and green circles, respectively. Images have been band-pass filtered to improve visualization of individual particles. Aggregation was seen in the presence of phosphocholine, and was exacerbated at pH 5. Few pentamers were visible at pH 5 in the presence of phosphocholine. Scale bar = 100 nm for all panels.

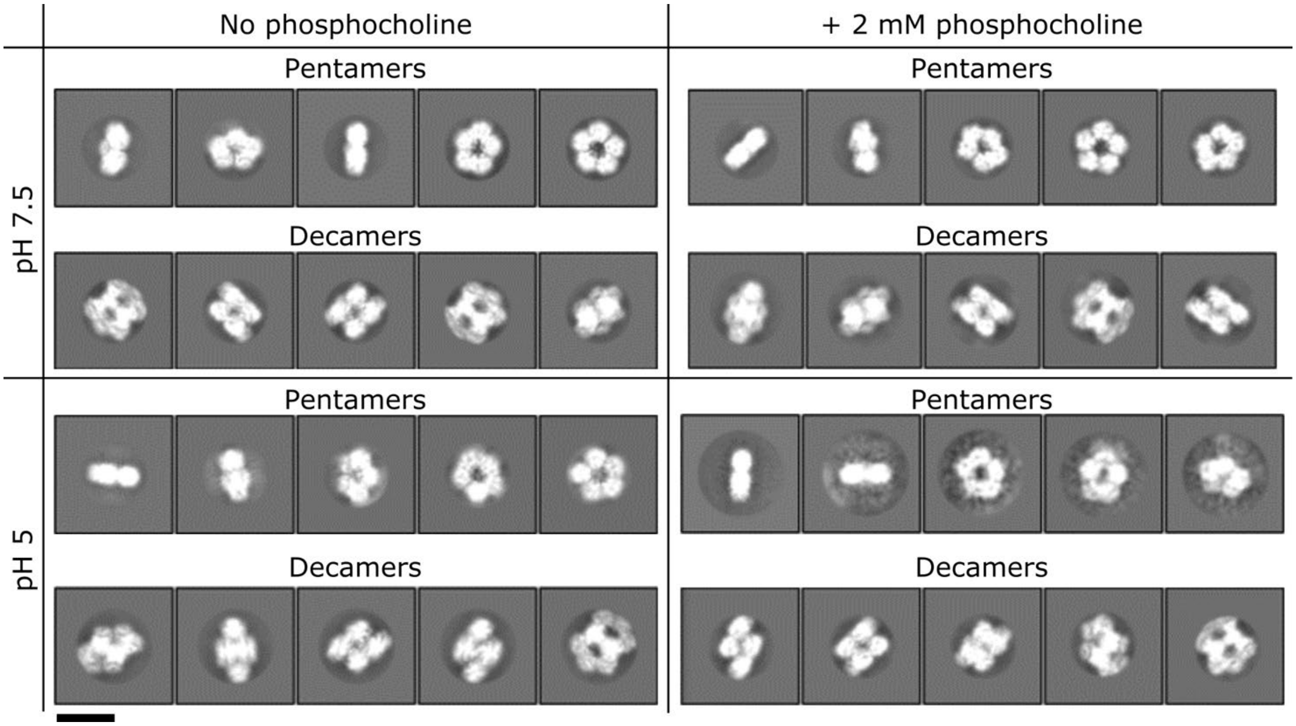

Figure S4. Representative classes of pentameric and decameric CRP for all 4 conditions. Scale bar = 10 nm for all classes.

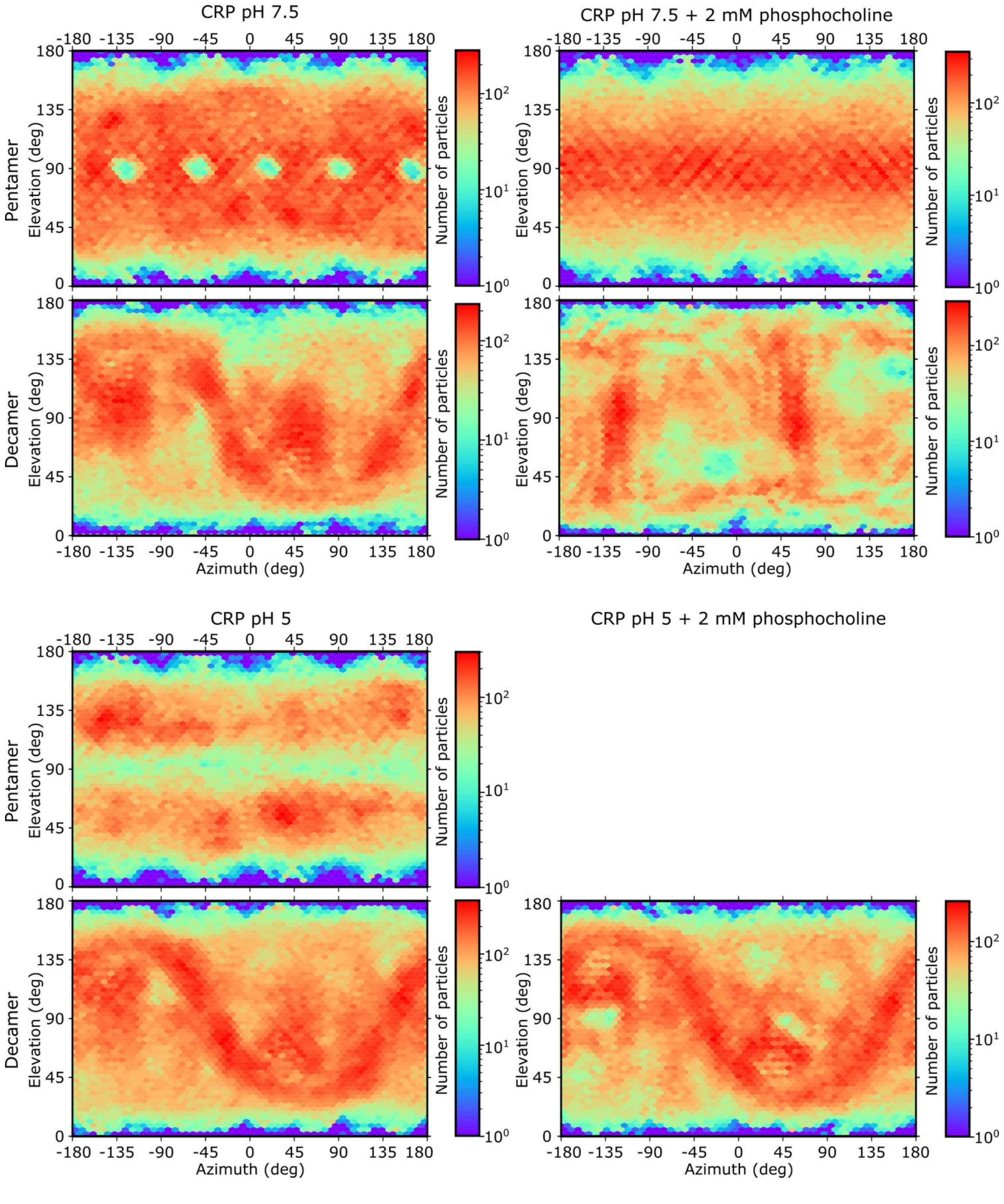

Figure S5. Euler plots showing angular distribution of particles used to reconstruct each 3D map for the conditions indicated. Plots for pentamers are for non-symmetrised (C1) reconstructions. Numbers of particles in each orientation is signified from low (blue) to high (red) according to the adjacent key. Plots were constructed using Angdist (2).

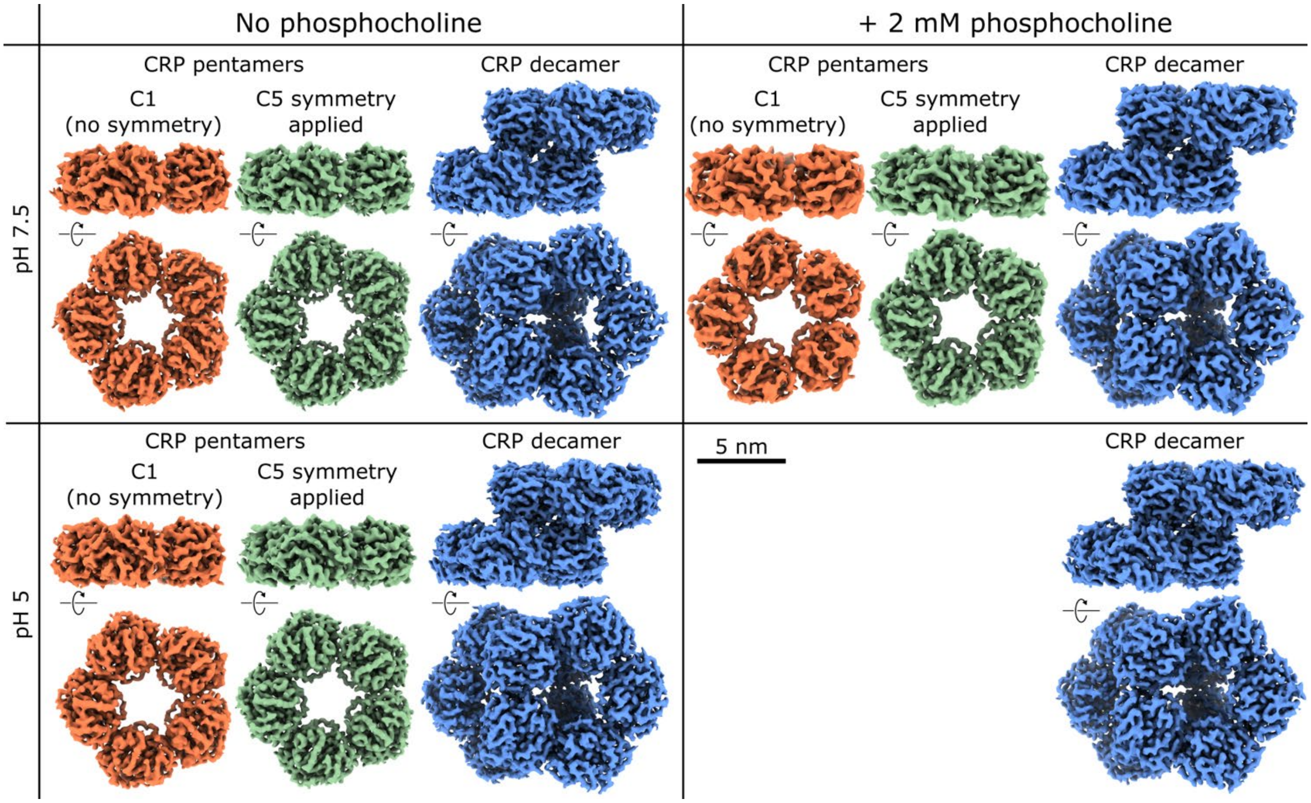

Figure S6. Overview of all 10 reconstructions determined for the 4 conditions indicated. Scale bar = 5 nm for all maps.

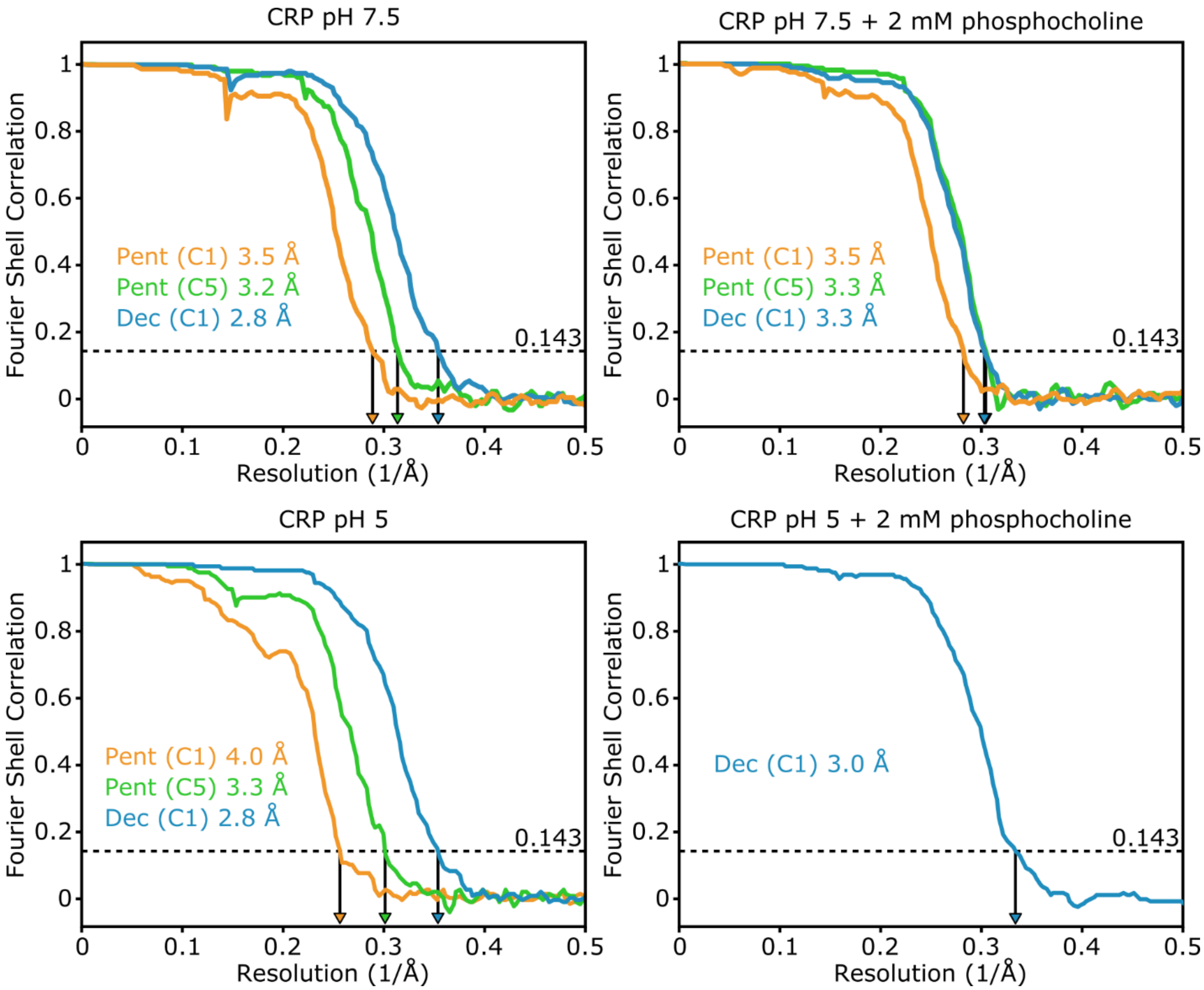

Figure S7. Fourier shell correlation (FSC) plots for each reconstruction. Plots are from gold-dstandard masked reconstructions. The overall resolution of the maps is dictated by the FSC = 0.143 threshold (dashed line) and denoted by coloured arrows. Pent and Dec are pentamer and decamer, respectively, with the symmetry applied in parentheses.

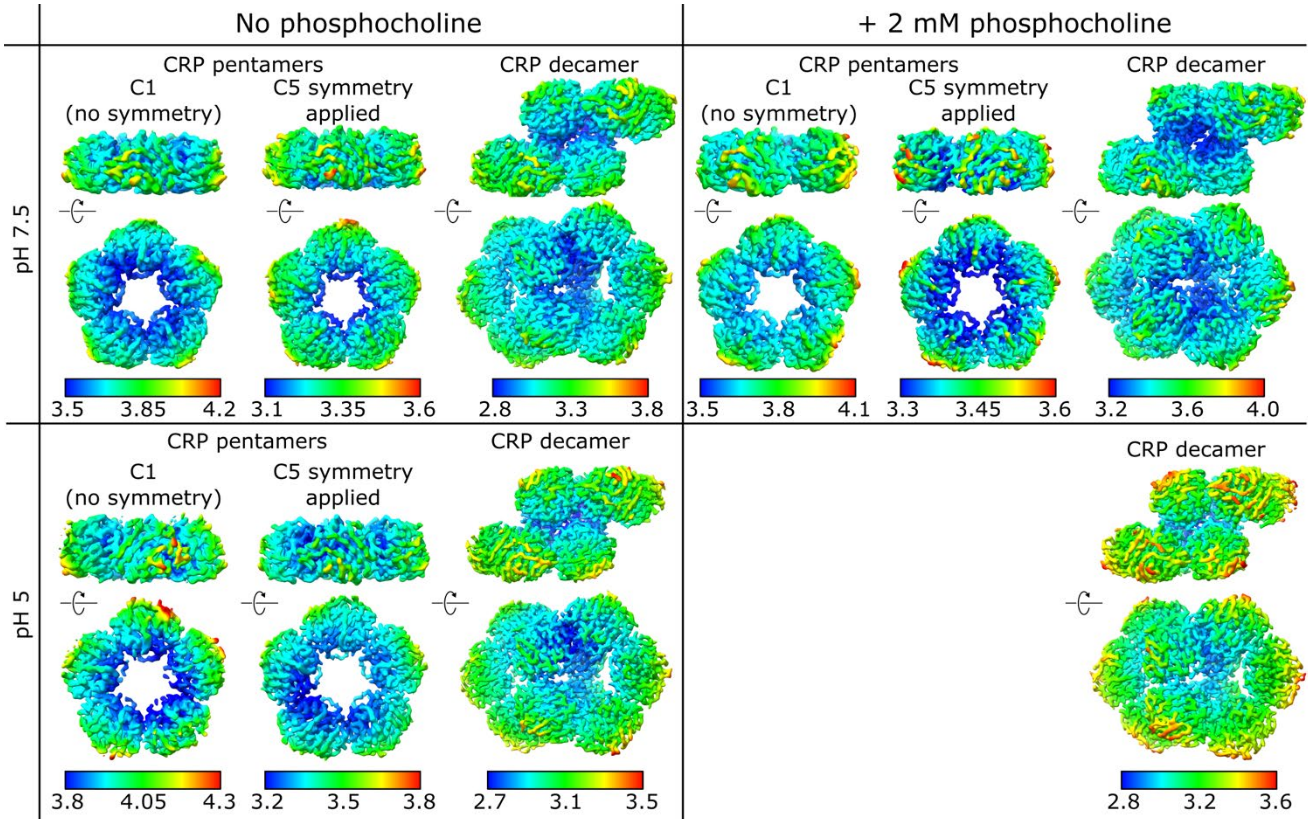

Figure S8. Local-resolution estimation of each reconstruction. Resolution in Ångstroms is indicated from good (blue) to poor (red) according to the corresponding colour keys.

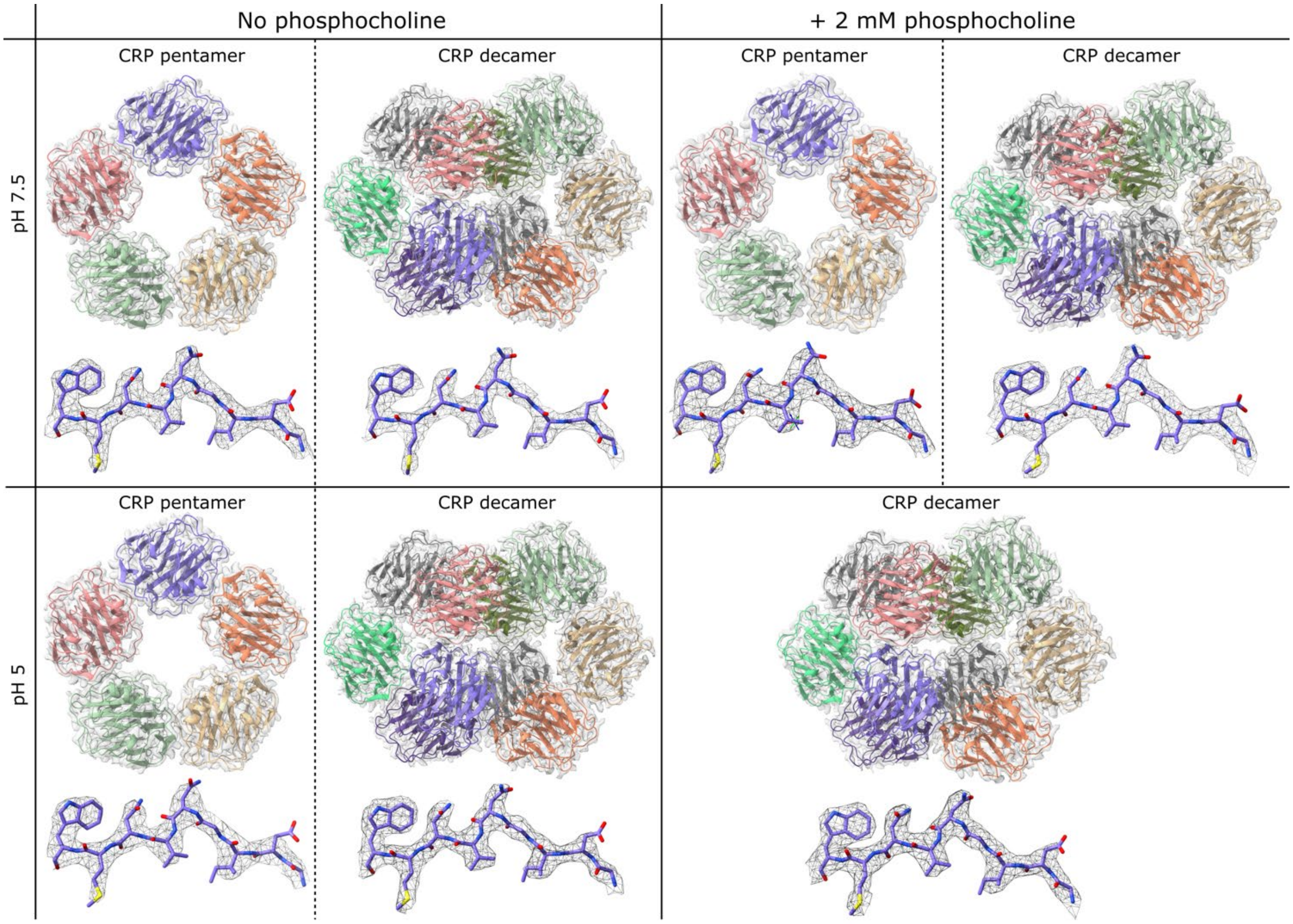

Figure S9. PDB models and sidechain densities for all conditions. CRP monomers are coloured by chain. EM map density is shown as grey for whole models and as mesh for sidechain densities. Carbon, nitrogen, oxygen and sulfur atoms are coloured purple, blue, red and yellow, respectively.

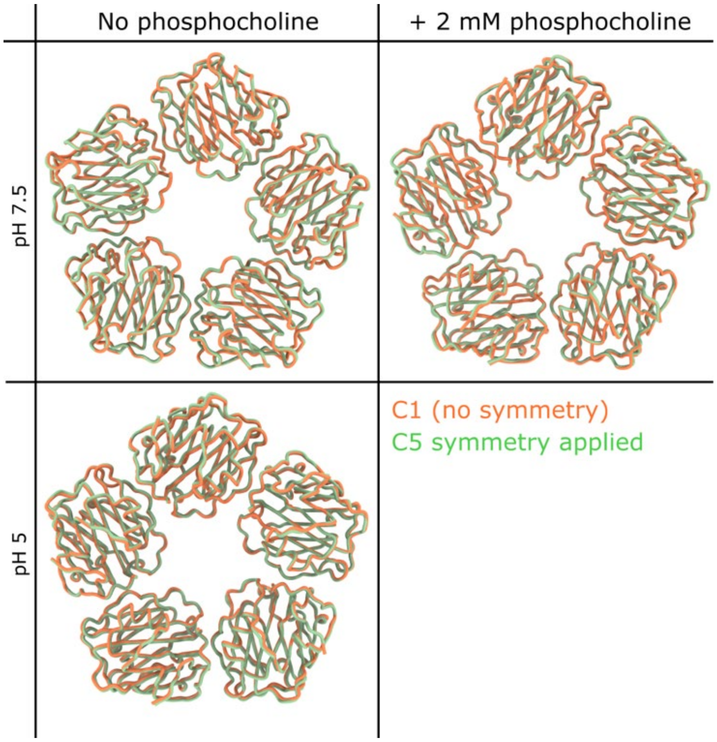

Figure S10. Comparing protein backbones of models built into either non-symmetrised or C5-symmetrised reconstructions of pentameric CRP.

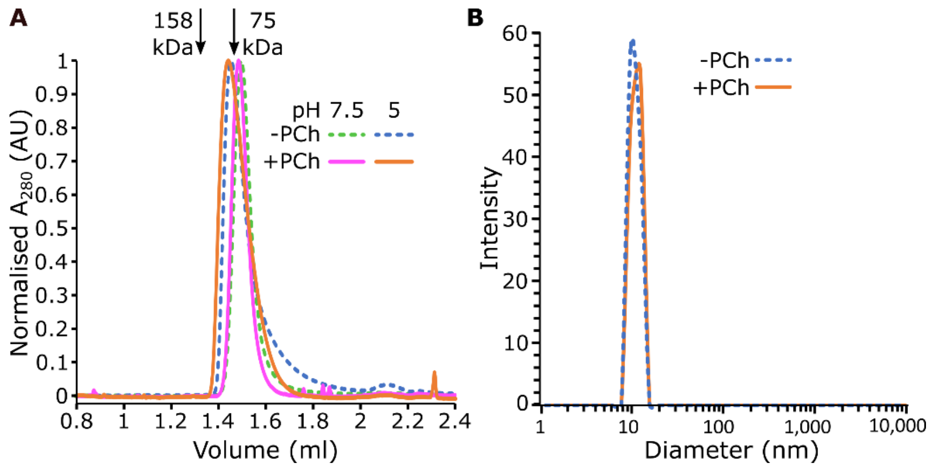

Figure S11. Size exclusion chromatography and dynamic light scattering profiles of CRP for each condition. A) Size exclusion chromatography (SEC) of the conditions tested under cryoEM (pH 7.5/5, +/- PCh 2mM). CRP was added to a superdex 200 increase 3.2/300 size exclusion column at 0.5 mg/ml, an acute phase concentration. Arrows indicate standards of the globular proteins Aldolase (pH 7.4, 158 kDa) and Conalbumin (pH 7.4, 75 kDa). **B)** Dynamic light scattering of ~2.8 mg/ml CRP at pH 5 ± PCh 2 mM with 0.05% tween. One representative singlet from a technical triplicate is shown.

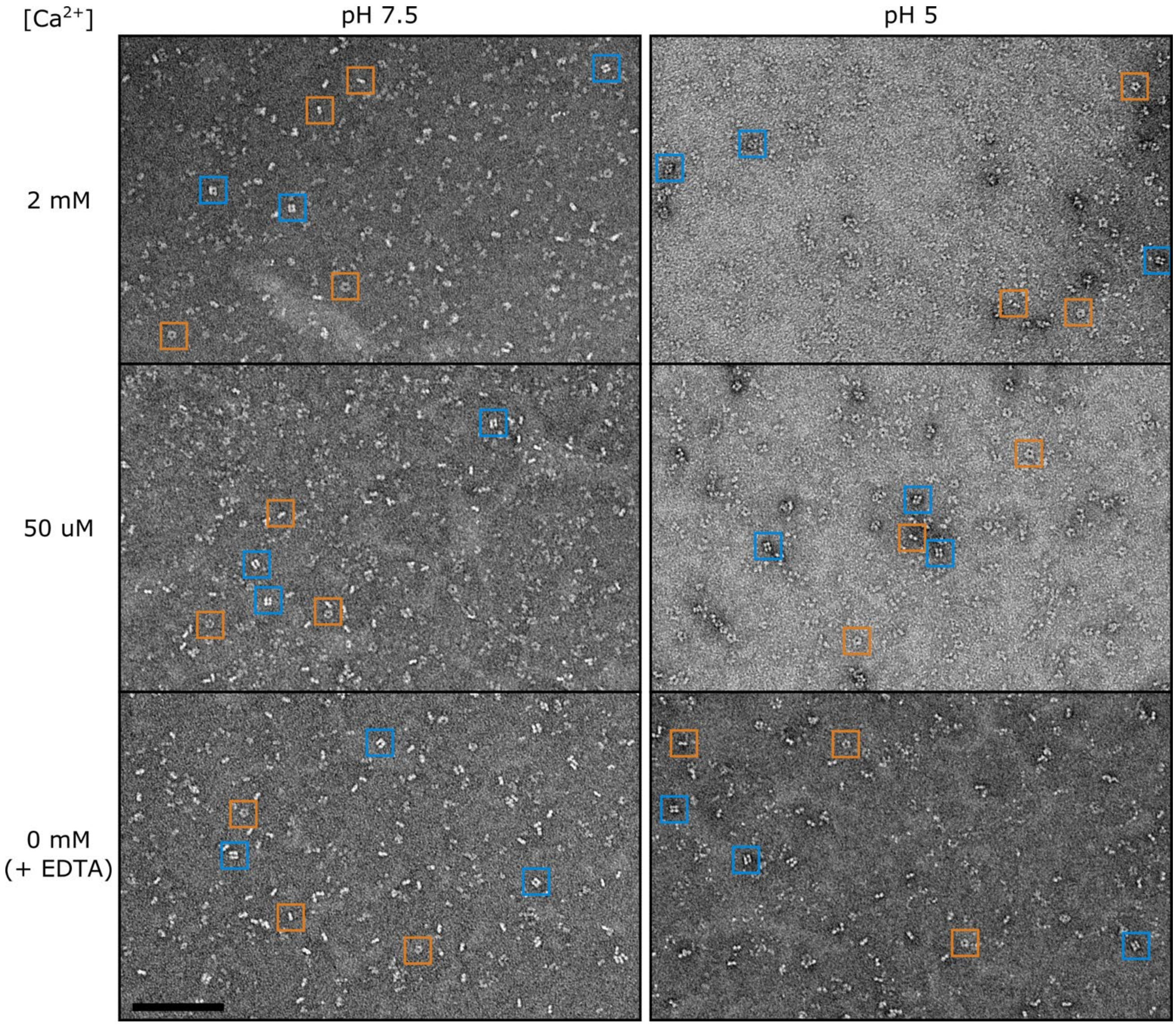

Figure S12. Negative stain EM of CRP under pH and calcium concentrations tested in ELISAs. Individual pentamers dominate (orange squares), but decamers are also present (blue squares). Scale bar = 100 nm for all panels.

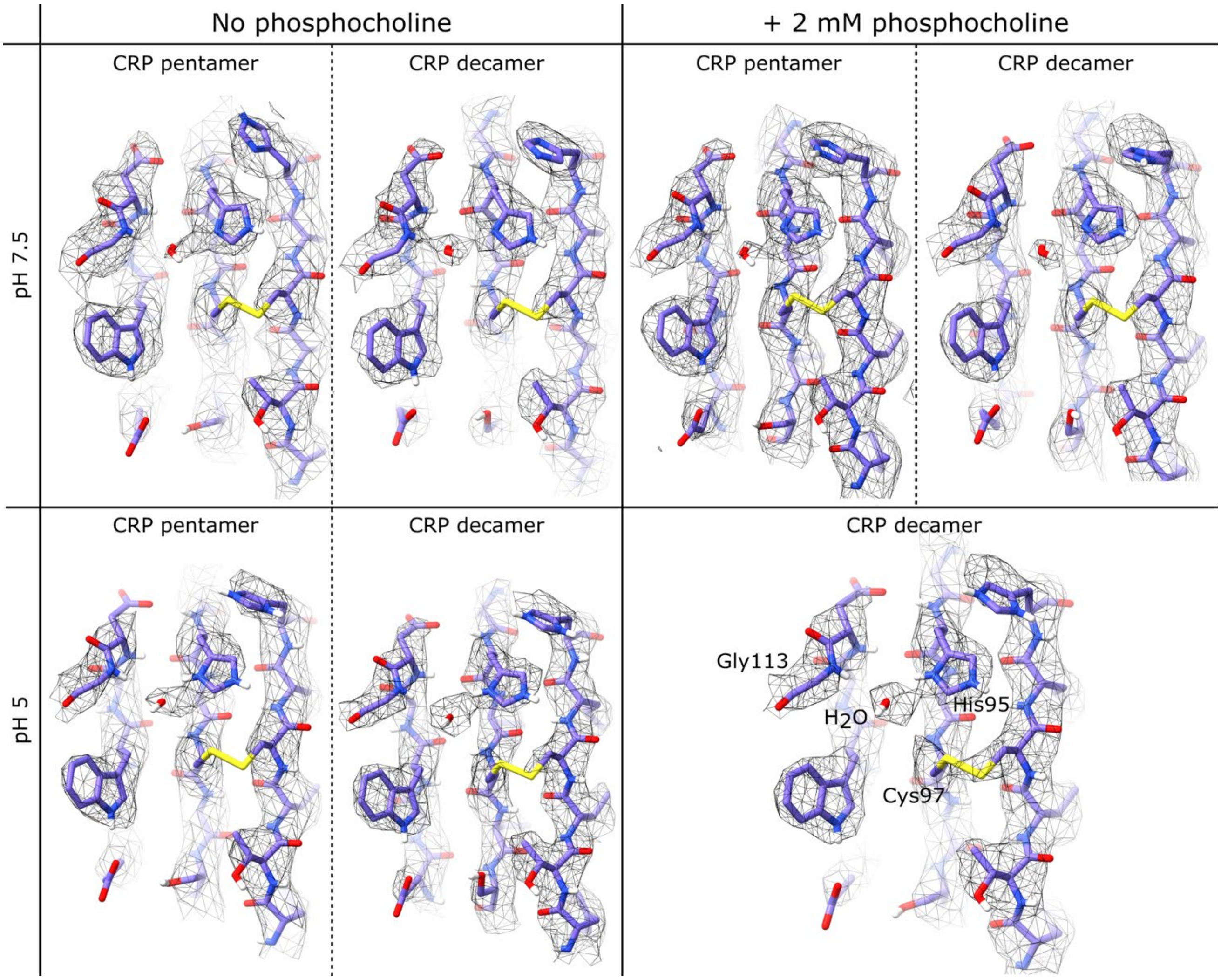

Figure S13. Analysis of additional density proximal to His95, Cys97 and Gly113. Density (black mesh) and chain A of each high-resolution model for all conditions. A water molecule modelled into each density can hydrogen bond to deprotonated (pH 7.5) and protonated (pH 5) histidine residue His95 and the backbone amine of glycine Gly113. Carbon, nitrogen, oxygen, sulphur and hydrogen atoms are coloured light purple, blue, red, yellow and white, respectively.

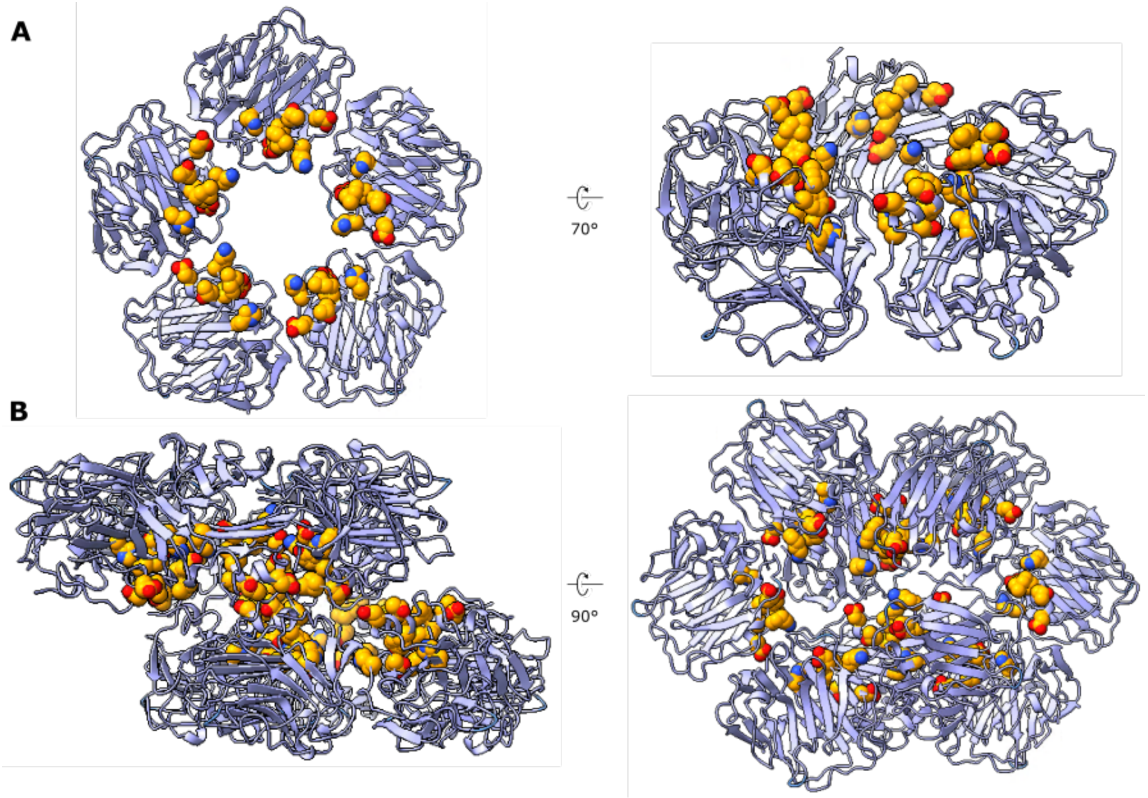

Figure S14. Locations of CRP residues involved in C1q binding and activation. Locations of residues previously known to be important in C1q binding and complement activation include His38, Lys57, Arg58, Glu88, Asp112, Lys114, Asp169, Thr173, Tyr175 and Leu176 (3-5). These residues are shown on the C5-symmetric pentameric model at pH 7.5 **(A)**. Model is in light blue, and the carbon atoms of highlighted residues are coloured orange, with the oxygen and nitrogen atoms coloured red and blue, respectively. **B)** Locations of the same residues on the decameric model at pH 7.5. Model is coloured light purple with the carbon atoms of highlighted residues and heteroatoms coloured as in **A**. For clarity, Lys57, Arg58 and Glu88 have not been shown.

3 Supplementary Tables

Model	Angles (°)					
	1	2	3	4	5	sd
**Dec pH 7.5**	108	108	108	108	109	0.4
**Dec pH 7.5**	108	108	108	108	107	0.4
**Dec pH 5**	108	108	108	109	107	0.7
**Dec pH 5**	108	108	110	109	107	1.1
**Pent C5 pH 7.5**	108	108	108	108	108	0.0
**Pent C5 pH 5**	108	108	108	108	108	0.0
**Pent C5 pH 7.5+PCh**	108	108	108	108	108	0.0
**Dec pH 7.5+PCh**	108	108	107	108	108	0.4
**Dec pH 7.5+PCh**	109	108	108	109	107	0.8
**Dec pH 5+PCh**	108	108	108	108	107	0.4
**Dec pH 5+PCh**	108	108	108	109	107	0.7
**Dec pH 7.5+PCh**	108	108	107	108	108	0.4

Table S1. Angles between monomers within high-resolution pentameric CRP structures as measured between Asp60 residues, in cryoEM derived atomic models presented in this study. Dec and Pent denote decameric and pentameric CRP structures, respectively. Red and blue signify larger and smaller angles, respectively. Angles were measured using UCSF Chimera.

PDB code	Pentamers in unit cell	Angles (°)					
		1	2	3	4	5	sd
**1B09**	1	105	108	108	105	112	2.9
**3L2Y**	4	107	109	106	109	108	1.3
		107	110	107	109	108	1.3
		108	108	107	109	108	0.7
		107	110	106	110	107	1.9
**3PVN**	4	107	110	107	108	108	1.2
		109	107	108	109	108	0.8
		110	108	107	110	110	1.4
		111	104	110	109	107	2.8
**3PVO**	4	109	106	110	108	107	1.6
		108	108	109	107	108	0.7
		109	108	108	109	106	1.2
**1GNH**	2	111	106	108	108	107	1.9
		109	106	108	107	108	1.1
**1LJ7**	2	108	109	107	109	109	0.9
		107	107	108	109	108	0.8
		107	109	108	106	109	1.3

Table S2. Angles between monomers within existing crystal structures of pentameric CRP as measured between Asp60 residues. Angles were measured using UCSF Chimera and were calculated for each of the CRP pentamers within each crystallographic unit cell.

Model 1	Model 2	Cross correlation	Global RMSD
Pent pH 7.5	Pent pH 5	0.9745	0.72
Pent pH 7.5+PCh	Pent pH 7.5	0.9719	0.41
Pent pH 7.5+PCh	Pent pH 5	0.9659	0.72
Dec pH 7.5	Dec pH 5	0.9509	0.84
Dec pH 7.5+PCh	Dec pH 5+PCh	0.9449	0.82
Dec pH 7.5+PCh	Dec pH 7.5	0.968	0.45
Dec pH 5+PCh	Dec pH 5	0.9772	0.6
Dec pH 7.5+PCh	Dec pH 5	0.9466	0.81
Dec pH 7.5	Dec pH 5+PCh	0.9551	0.87
Pent pH 7.5	Dec pH 7.5	0.9597	0.72
Pent pH 7.5	Dec pH 5	0.9538	0.71
Pent pH 7.5	Dec pH 7.5+PCh	0.9617	0.7
Pent pH 7.5	Dec pH 5+PCh	0.964	0.75
Pent pH 5	Dec pH 7.5	0.9524	0.79
Pent pH 5	Dec pH 5	0.9453	0.68
Pent pH 5	Dec pH 7.5+PCh	0.9602	0.78
Pent pH 5	Dec pH 5+PCh	0.957	0.71
Pent pH 7.5+PCh	Dec pH 7.5	0.9489	0.75
Pent pH 7.5+PCh	Dec pH 5	0.9399	0.74
Pent pH 7.5+PCh	Dec pH 7.5+PCh	0.9688	0.7
Pent pH 7.5+PCh	Dec pH 5+PCh	0.9585	0.77

Table S3. Cross correlation and RMSD comparisons between each high-resolution model and map resolved from cryoEM data presented in this study. Cross correlation and RMSD values were calculated using chimera. Pentamers (Pent) were compared to chains A-E of decamers (Dec). Red signifies greater deviation between maps/models, whereas blue represents greater similarities. Cross correlation and RMSD values were calculated using UCSF Chimera.

Pentameric model at:	PDB code	Global RMSD
pH 7.5	1B09	1.40
pH 7.5	3PVO	0.94
pH 7.5	3L2Y	0.92
pH 7.5	3PVN	1.08
pH 7.5	1GNH	1.31
pH 5	1B09	1.50
pH 5	3PVO	0.98
pH 5	3L2Y	0.91
pH 5	3PVN	1.10
pH 5	1GNH	1.37
pH 7.5+PCh	1B09	1.40
pH 7.5+PCh	3PVO	0.95
pH 7.5+PCh	3L2Y	0.92
pH 7.5+PCh	3PVN	1.09
pH 7.5+PCh	1GNH	1.32

Table S4. RMSD between C5-symmetric pentameric models resolved from cryoEM data presented in this study with a subset of crystal structures. Red signifies greater deviation between models, whereas blue represents greater similarities. RMSD values were calculated using UCSF Chimera.

Model	Oligomeric state	pH	Ligand
1B09	Pentamer	7.6	PCh
3LY2	Decamer	7.5	PE
3PVN	Decamer	8	Zn^{2+} not Ca^{2+}
3PVO	Decamer	8	
1GNH	Pentamer	7.7	
1LJ7	Decamer	8.2	
pH 7.5	Pentamer	7.5	
pH 5	Pentamer	5	
pH 7.5	Pentamer	7.5	PCh
pH 7.5	Decamer	7.5	
pH 5	Decamer	5	
pH 7.5	Decamer	7.5	PCh
pH 5	Decamer	5	PCh

Table S5. Comparison of EM derived and X-ray crystallographic protein models. X-ray crystal structures are denoted by their PDB codes and the cryoEM derived models presented in this study. Oligomeric state, pH and ligands present are shown. Red and blue signify more acidic and more basic pH, respectively.

Supplemental references

1. S. H. W. Scheres. Amyloid structure determination in RELION-3.1. *Acta Crystallographica Section D, Structural Biology* (2020) 76(1):94-101. doi: https://doi.org/10.1107/S2059798319016577.

2. G. Gaullier. Angdist: Plot the 2D histogram of Euler angles covered by a set of cryo-EM particles (v1.2). *Zenodo* (2020). doi: https://doi.org/10.5281/zenodo.4395763.

3. A. Agrawal, J. E. Volanakis. Probing the C1q-binding site on human C-reactive protein by site-directed mutagenesis. *The Journal of Immunology* (1994) 1(152):5404-10.

4. A. Agrawal, A. K. Shrive, T. J. Greenhough, J. E. Volanakis. Topology and structure of the C1q-binding site on C-reactive protein. *Journal of Immunology* (2001) 166(6):3998-4004. doi: https://doi.org/10.4049/jimmunol.166.6.3998.

5. R. Bang, L. Marnell, C. Mold, M.-P. Stein, K. T. Du Clos, C. Chivington-Buck, et al. Analysis of Binding Sites in Human C-reactive Protein for FcγRI, FcγRIIA, and C1q by Site-directed Mutagenesis. *The Journal of Biological Chemistry* (2005) 280(26):25095-102. doi: https://doi.org/10.1074/jbc.M504782200.
